# Effects of different exercises on improving gait performance in patients with Parkinson’s disease: a systematic review and network meta-analysis

**DOI:** 10.3389/fnagi.2025.1496112

**Published:** 2025-02-26

**Authors:** Ying Li, Jieling Huang, Jinguo Wang, Yue Cheng

**Affiliations:** ^1^College of Sports Science, Jishou University, Jishou, China; ^2^Faculty of Health Sciences and Physical Education, Macao Polytechnic University, Macao, Macao SAR, China; ^3^Department of Neurology, People’s Hospital of Changshou, Chongqing, China

**Keywords:** Parkinson’s disease, gait, 6MWT, network meta-analysis, systematic review

## Abstract

**Objective:**

Gait disorder represents a characteristic symptom of Parkinson’s disease (PD), and exercise has been established as an effective intervention for gait management in PD. However, the relative efficacy of various exercise types in improving gait among PD patients remains unclear. This study aimed to compare the effectiveness of different movement-based interventions in enhancing gait for individuals with PD through a network meta-analysis.

**Methods:**

A comprehensive search was conducted across multiple databases, including PubMed, Cochrane Library, Embase, Web of Science, and CNKI. The methodological quality of included studies was evaluated using the Cochrane Bias risk tool. Data was extracted from these studies to compare the efficacy of 29 distinct exercise interventions on gait performance in patients with PD.

**Results:**

The analysis encompassed 68 randomized controlled trials (RCTs), involving a total of 3,114 participants. The results of the network meta-analysis showed that DE is higher than CON (SMD, 2.11; 95% CI 1.07 to 3.15), WE (SMD, 2.16; 95% CI 0.90 to 3.43), HE (SMD, 2.19; 95% CI 0.95 to 3.44), OE (SMD, 2.66; 95% CI 1.16 to 4.16), TR (SMD, 2.62; 95% CI 1.45 to 3.79) to better improve Gait velocity in patients with Parkinson’s disease. DE is superior to CON (SMD, 2.08; 95% CI 0.04 to 4.13) in improving Step length. FAE is superior to CON (SMD, 1.01; 95% CI 0.04 to 1.98), BDJ (SMD, 1.20; 95% CI 0.15 to 2.25), RAGT (SMD, 1.29; 95% CI 0.07 to 2.52), DE (SMD, 1.57; 95% CI 0.36 to 2.77), TR (SMD, 1.62; 95% CI 0.48 to 2.76), OE (1.76, 95% CI 0.57 to 2.94) in improving Gait velocity. RAGT is superior to CT (MD, 2.02; 95% CI 0.41 to 3.63), TR (MD, 2.51; 95% CI 1.17 to 3.84), AE (MD, 2.66; 95% CI 0.45 to 4.88), BDJ (MD, 2.77; 95% CI 0.93 to 4.61), CON (MD, 2.83; 95% CI 1.30 to 4.36), DTT (MD, 12.84; 95% CI 10.05 to 15.63) in improving 6MWT.

**Conclusion:**

Our study found that DE improved gait speed and step length in patients with Parkinson’s disease better than other forms of exercise. FAE and RAGT were more effective than other exercises in improving step length and 6MWT in patients with Parkinson’s disease.

## Introduction

PD is the second most prevalent neurodegenerative disorder characterized by motor activity deterioration, resulting from damage to the dopaminergic nigrostriatal system (Pajares et al., 2020; Simon et al., 2020). The incidence of PD correlates directly with age (Hallett et al., 2019). PD diagnoses are projected to double from 6 million in 2015 to 12 million in 2040, coinciding with approximately 1% of the global population exceeding 60 years of age (Ascherio and Ma, 2016; Tysnes and Storstein, 2017; Dorsey and Bloem, 2018). Gait abnormality represents a primary cause of motor dysfunction in PD patients (Frazzitta et al., 2009). These gait abnormalities manifest primarily as postural irregularities, diminished muscle strength in major limb and trunk muscle groups, reduced balance function, initiation difficulties, decreased stride length, inability to halt at will, turning challenges, and bradykinesia. This walking dysfunction in PD patients elevates fall risk and significantly impairs daily living capabilities.

Current PD treatment strategies aim to alleviate symptoms and decelerate disease progression (Raza et al., 2019). The prevailing PD treatment model involves anti-Parkinson’s medications or Deep Brain Stimulation, which often prove inadequate, yielding minimal or no long-term patient improvement (Scelzo et al., 2019). Adjunctive and complementary treatments, such as exercise, can mitigate side effects of anti-Parkinson’s drug therapy and have demonstrated efficacy in improving movement disorders including balance, gait, fall risk, and physical function while reducing fall incidence (Feng et al., 2020; Mak and Wong-Yu, 2019). An increasing body of evidence supports the crucial role of exercise therapy (Tomlinson et al., 2013; Abbruzzese et al., 2016). Dance exercise (DE) can down-regulate *α*-Syn protein level to a certain extent, inhibit neuronal apoptosis, improve mitochondrial dysfunction, and thus restore the motor function of early patients (Koo et al., 2017). Resistance training (RT) is a strength exercise that can enhance muscle strength by overcoming resistance in local muscle groups. It can also improve the body’s neuroplasticity, up-regulate the expression level of dopaminergic neurotransmitters and receptors, promote the release of non-dopaminergic transmitters such as norepinephrine and 5-hydroxytryptamine (Qian et al., 2024), and improve the motor symptoms of Parkinson’s patients. Aerobic exercise (AE) can improve motor function in patients with Parkinson’s disease by regulating neurotrophic factors to support synaptic formation and angiogenesis, inhibit oxidative stress and improve mitochondrial function (Feng et al., 2020). However, the optimal exercise type for treating gait in PD patients remains unclear. According to Jiang’s traditional meta-analysis, Robotic Assisted Gait Training (RAGT) can effectively enhance PD patients’ walking function and gait performance (Jiang et al., 2024). In a conventional meta-analysis, Bishnoi found that Treadmill Training improved step and stride lengths in PD patients (Bishnoi et al., 2022). Nevertheless, a direct comparison between RAGT and Treadmill Training is currently lacking.

Network meta-analysis (NMA) has gained prominence in evaluating medical interventions due to its capacity to estimate the relative effectiveness and ranking of interventions, even in the absence of direct comparisons (Bafeta et al., 2014). Although Yang explored the influence of exercise patterns on Parkinson’s patients, the exercise was classified into 24 categories, and there was a lack of research on six-minute walk test (6MWT) and Step-length outcome indicators (Yang et al., 2022). Victor explored the intervention measures to improve gait in Parkinson’s disease, but there was a lack of studies on Stride length, 6MWT, and Step length (Hvingelby et al., 2022). Therefore, our study made a detailed division of movement modes, compared the gait forms of more PD patients, determined the best exercise mode to improve the gait of PD patients, and guided PD patients to choose the best exercise mode.

## Methods and analysis

### Registration

This network meta-analysis was designed according to the guidelines for Preferred Reporting Items of Systems Review and Network Meta-Analysis (PRISMA-NMA) (Hutton et al., 2015).

### Search strategy

The computer searched PubMed, Web of Science, Embase, Cochrane Library, CNKI, and other databases, and the search period was established until August 20, 2024. The search takes the way of combining subject words and free words. The search strategy uses Pubmed as an example, as shown in Appendix 1.

### Study selection

The inclusion criteria for study selection were based on the PICOS methodology (Participants, interventions, comparators, outcomes, and study design) (Hutton et al., 2015), shown in Table 1. In addition, we provide detailed definitions of 29 intervention terms. Each intervention is defined in Appendix 2.

**Table 1:** Inclusion and exclusion criteria.

Category	Inclusion criteria	Exclusion criteria
Population	Parkinson’s disease was diagnosed in patients >18 years of age	Patients with severe comorbidities such as severe hypertension, heart disease, or other serious systemic diseases
Interventions	Aerobic exercise (AE), Aquatic Exercise (AQE), Whole body vibration training (WBV), Virtual reality (VR), Treadmill training (TT), Resistance training (RT), Tai Chi (TC), Power Training (PT), Biofeedback Balance and Gait Training (BGT), Walking exercise (WE), Dance exercise (DE), Balance training (BT), Game training (GT), Baduanjin (BDJ), Home exercise (HE), Yoga (YG), Boxing exercise (BE), Robotic Assisted Gait Training (RAGT), Combined therapy (CT), Dual task training (DTT), Stretch exercise (SE), Five animal exercises (FAE), Other exercise (OE), Fitness exercise (FE), Qigong (QG), Virtual reality balance training (VRB), Core strength training (CST).	
Comparisons	Traditional Rehabilitation (TR), Control group (CON)	
Outcomes	Stride length, Gait velocity, 6 Minutes Walking Test (6MWT), Step length	
Study	Randomized controlled trial; published in English or Chinese	

### Data extraction

The following data were extracted independently by two reviewers: first author, year of publication, country, sample size, intervention mode, intervention time, and intervention period. Data are expressed as mean ± standard deviation (mean ± SD). If the outcome indicator reports multiple points, we extract data for the most recent time.

### Risk of bias assessment

The risk of bias was assessed independently by two reviewers and by a third reviewer using the tools provided by the Cochrane Collaboration (Higgins et al., 2011), including sequence generation, hidden assignment, blinking, incomplete outcome data, non-selective reporting of results, and other sources of bias. Each criterion was judged to have a low, unclear, or high risk of bias.

### Data analysis

The netmeta package of R-4.2.1 software was used to perform mesh meta-analysis. Use the STATA 15.1 “networkplot” feature to draw and generate a network diagram that describes and presents different forms of exercises. We use nodes representing various interventions and edges representing head-to-head comparisons between interventions. Node splitting assesses inconsistencies between direct and indirect comparisons (Rücker and Schwarzer, 2015). The combined estimates and their 95% confidence intervals (95% CI) were calculated using random effects network analysis. When we are interested in results using the same unit of measure in a study, consider analyzing the results as a therapeutic effect by means difference (MD) or evaluating standardized mean difference (SMD). A pair-to-pair random-effects meta-analysis was used to compare various exercise therapies. The heterogeneity of all pair-to-pair comparisons was assessed using the *I^2^* statistic, and publication bias was evaluated using the *p*-value of Egger’s test and the funnel plot.

## Results

### Literature selection

**Figure 1:**
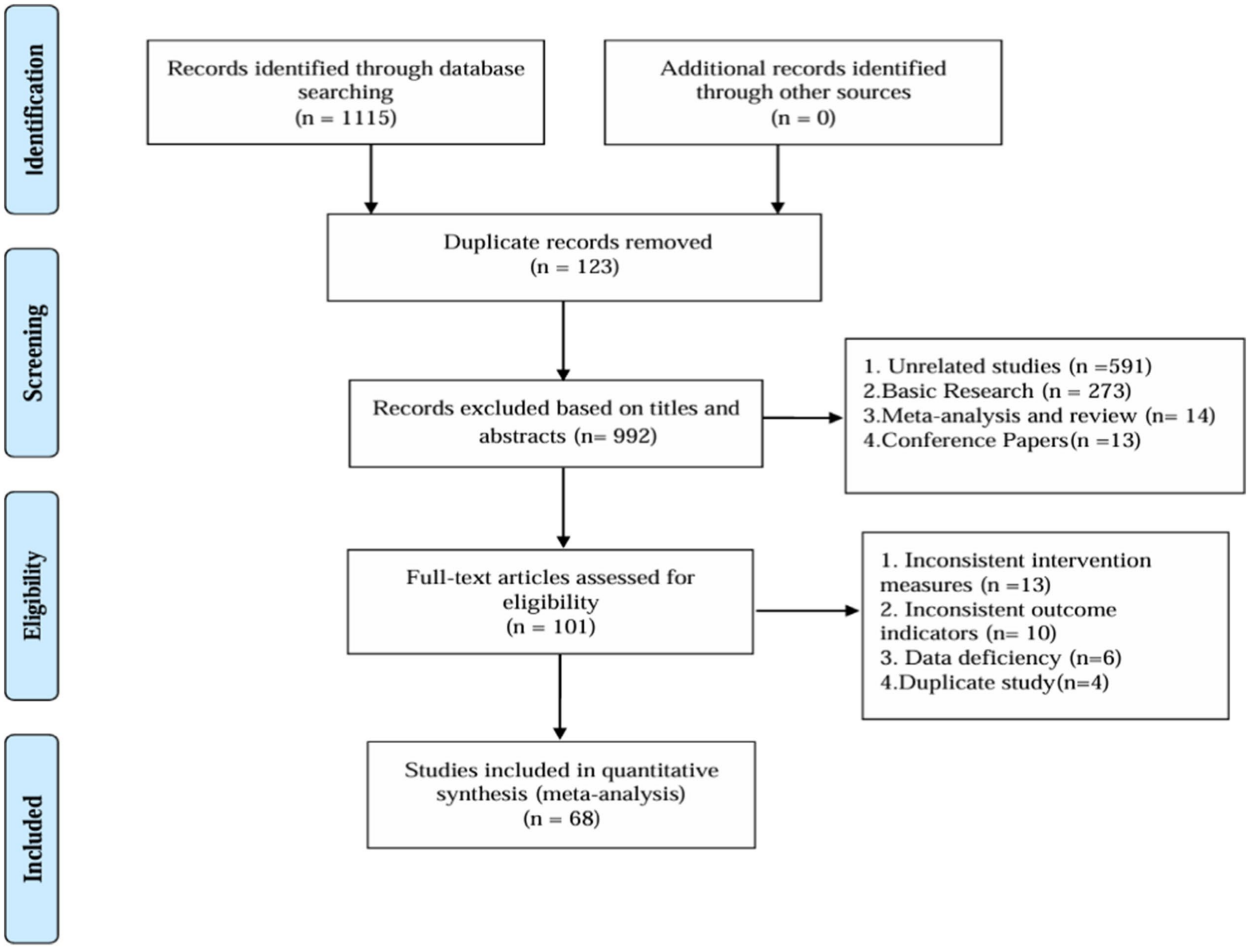
Flow chart.

After deleting duplicates, 1,115 records were retrieved, 123 duplicates were removed, 891 articles with inconsistent titles were deleted, 33 articles with inconsistent titles were removed after reading the full text, and 68 articles were finally included (Appendix 3). The research flow chart is shown in Figure 1.

### Study and participant characteristics

**Figure 2:**
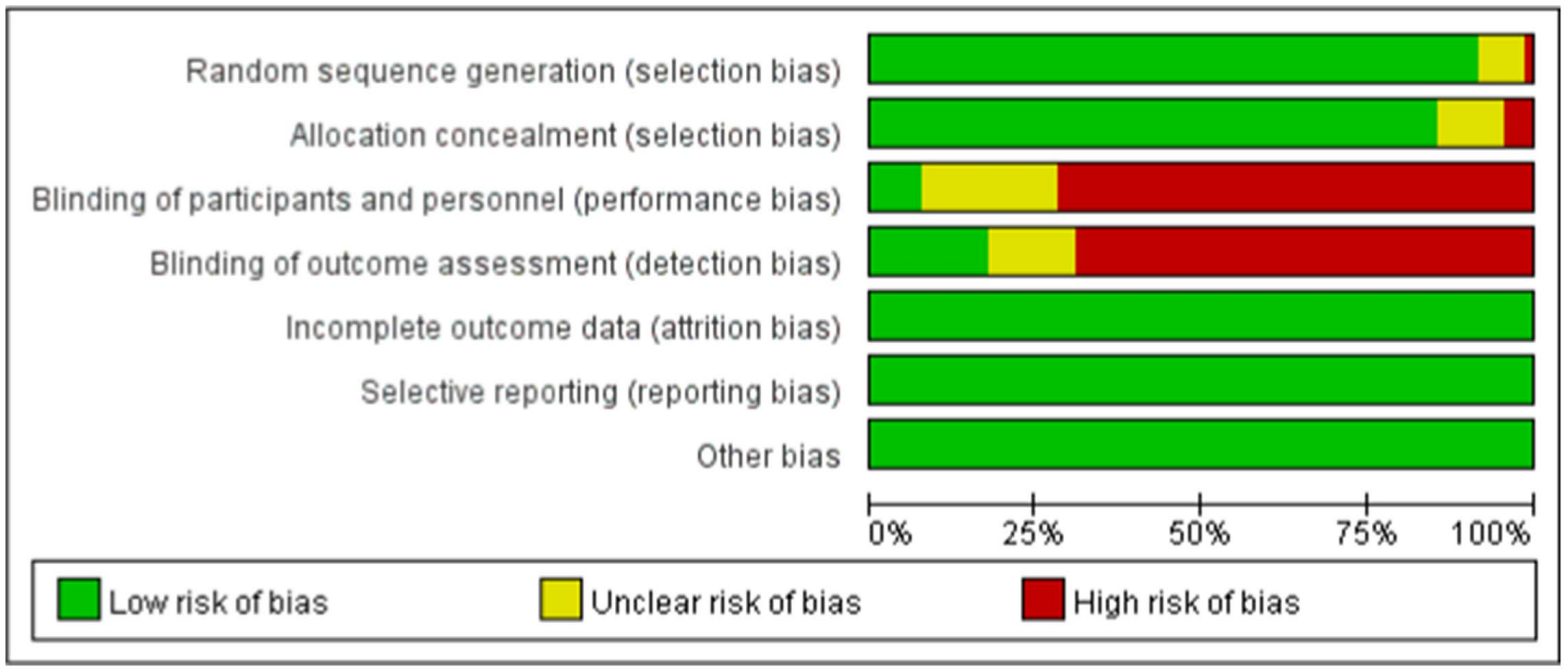
Percentage of studies examining the efficacy of exercise training in patients with non-specific chronic low back pain with low, unclear, and high risk of bias for each feature of the Cochrane risk of bias tool.

The included studies, published between 2007 and 2024, compared the effects of 29 different forms of exercise on people with Parkinson’s disease. The duration of intervention ranged from 3 to 48 weeks. A total of 3,114 patients were reported. Of all the included studies, 33 reported Gait velocity, 16 reported Step length, 19 reported Stride length and 35 reported 6MWT. The characteristics of the studies and participants are shown in Table 2 and Appendix 3. The risk of bias assessment for each study is summarized in Appendix 4 and Figure 2.

**Table 2:** General characteristics of patients.

Characteristics	Gait velocity	Step length	Stride length	6MWT
Publication characteristics				
Total number of unique studies included	33	15	19	35
Publication year				
2001–2010	2	0	3	2
2011–2020	18	6	10	23
2021–2024	13	9	6	10
Study design characteristics				
Range of study sample size				
1–50	22	7	15	23
51–100	10	8	4	9
101–150	1	0	0	3
151–200	0	0	0	0
No. of intervention arms included				
2	32	15	17	31
3	1	0	2	4
No. of studies containing the following treatment nodes				
Aerobic exercise (AE)	1	0	1	1
Aquatic Exercise (AQE)	0	0	0	3
Whole body vibration training (WBV)	0	0	0	1
Virtual reality (VR)	2	1	0	0
Treadmill training (TT)	1	0	3	5
Resistance training (RT)	0	0	0	2
Tai Chi (TC)	3	1	2	5
Power Training (PT)	2	0	0	0
Biofeedback Balance and Gait Training (BGT)	1	0	0	0
Control group (CON)	16	4	11	16
Walking exercise (WE)	2	0	3	5
Dance exercise (DE)	1	1	1	6
Balance training (BT)	4	3	0	1
Game training (GT)	0	0	0	1
Baduanjin (BDJ)	2	1	3	2
Home exercise (HE)	2	0	0	0
Yoga (YG)	0	0	1	0
Boxing exercise (BE)	1	0	0	1
Robotic Assisted Gait Training (RAGT)	1	1	1	2
Combined therapy (CT)	2	2	1	6
Traditional Rehabilitation (TR)	9	9	2	11
Dual task training (DTT)	3	2	3	1
Stretch exercise (SE)	2	1	2	2
Five animal exercises (FAE)	2	0	1	0
Other exercise (OE)	2	0	2	2
Fitness exercise (FE)	0	0	0	1
Qigong (QG)	6	3	3	0
Virtual reality balance training (VRB)	1	1	0	0
Core strength training (CST)	1	1	0	0
Time of intervention				
Unclear	1	0	1	1
3 weeks	2	1	1	0
4 weeks	3	3	3	3
5 weeks	1	0	1	0
6 weeks	2	1	3	2
8 weeks	5	3	1	7
10 weeks	1	1	0	5
11 weeks	1	0	0	0
12 weeks	13	4	7	12
16 weeks	1	1	1	2
24 weeks	2	1	1	3
48 weeks	1	0	0	0

### Gait velocity

**Figure 3:**
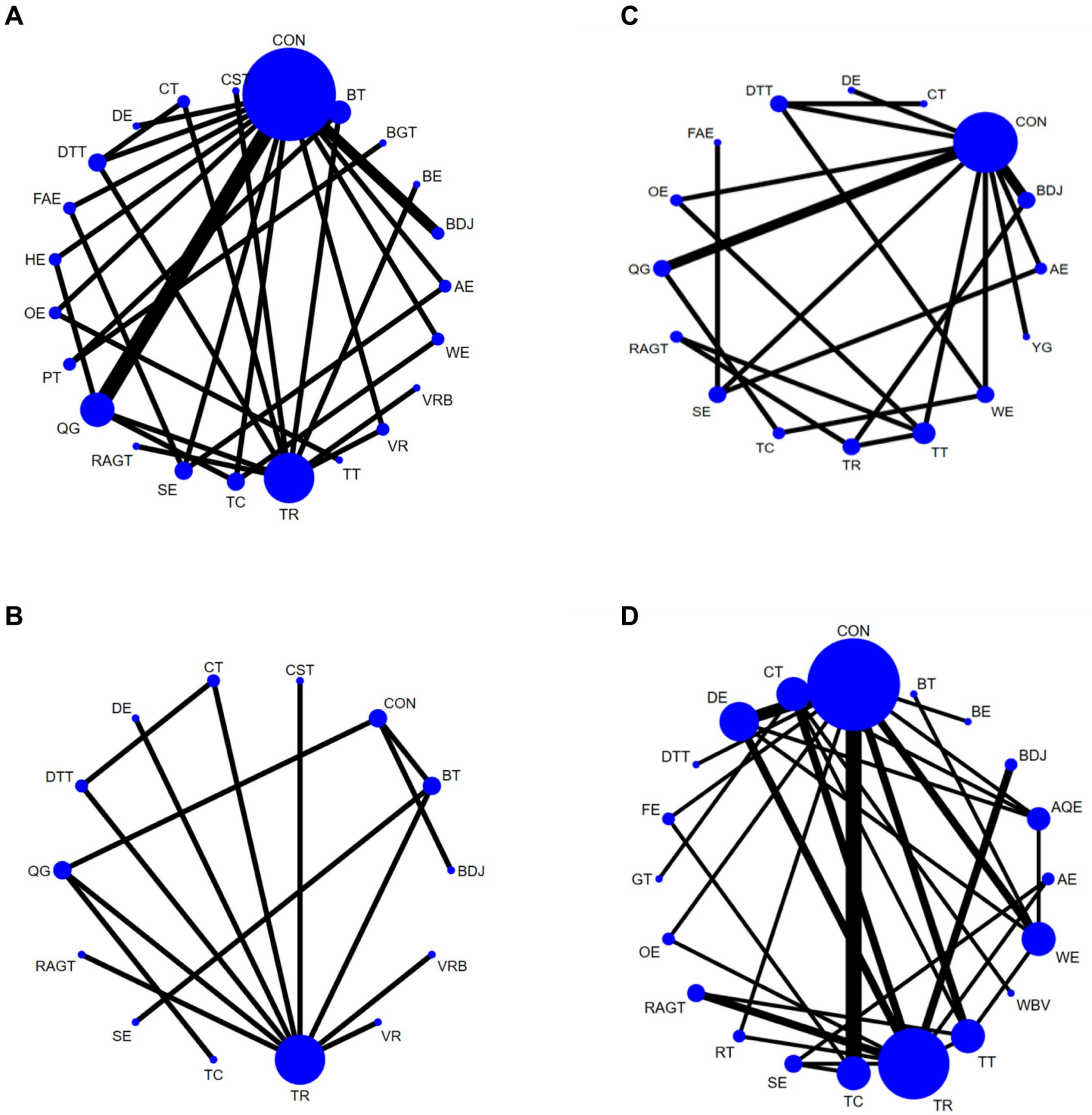
Network diagram of Gait velocity **(A)**, Step length **(B)**, Gait velocity **(C)**, and 6MWT **(D)**, in patients with Parkinson’s disease. The node size represents the number of times the exercise appears in any comparison about that treatment, and the width of the edge represents the total sample size in the comparison it connects. Aerobic exercise (AE), Aquatic Exercise (AQE), Whole body vibration training (WBV), Virtual reality (VR), Treadmill training (TT), Resistance training (RT), Tai Chi (TC), Power Training (PT), Biofeedback Balance and Gait Training (BGT), Control group (CON), Walking exercise (WE), Dance exercise (DE), Balance training (BT), Game training (GT), Baduanjin (BDJ), Home exercise (HE), Yoga (YG), Boxing exercise (BE), Robotic Assisted Gait Training (RAGT), Combined therapy (CT), Traditional Rehabilitation (TR), Dual task training (DTT), Stretch exercise (SE), Five animal exercises (FAE), Other exercise (OE), Fitness exercise (FE), Qigong (QG), Virtual reality balance training (VRB), Core strength training (CST).

**Figure 4:**
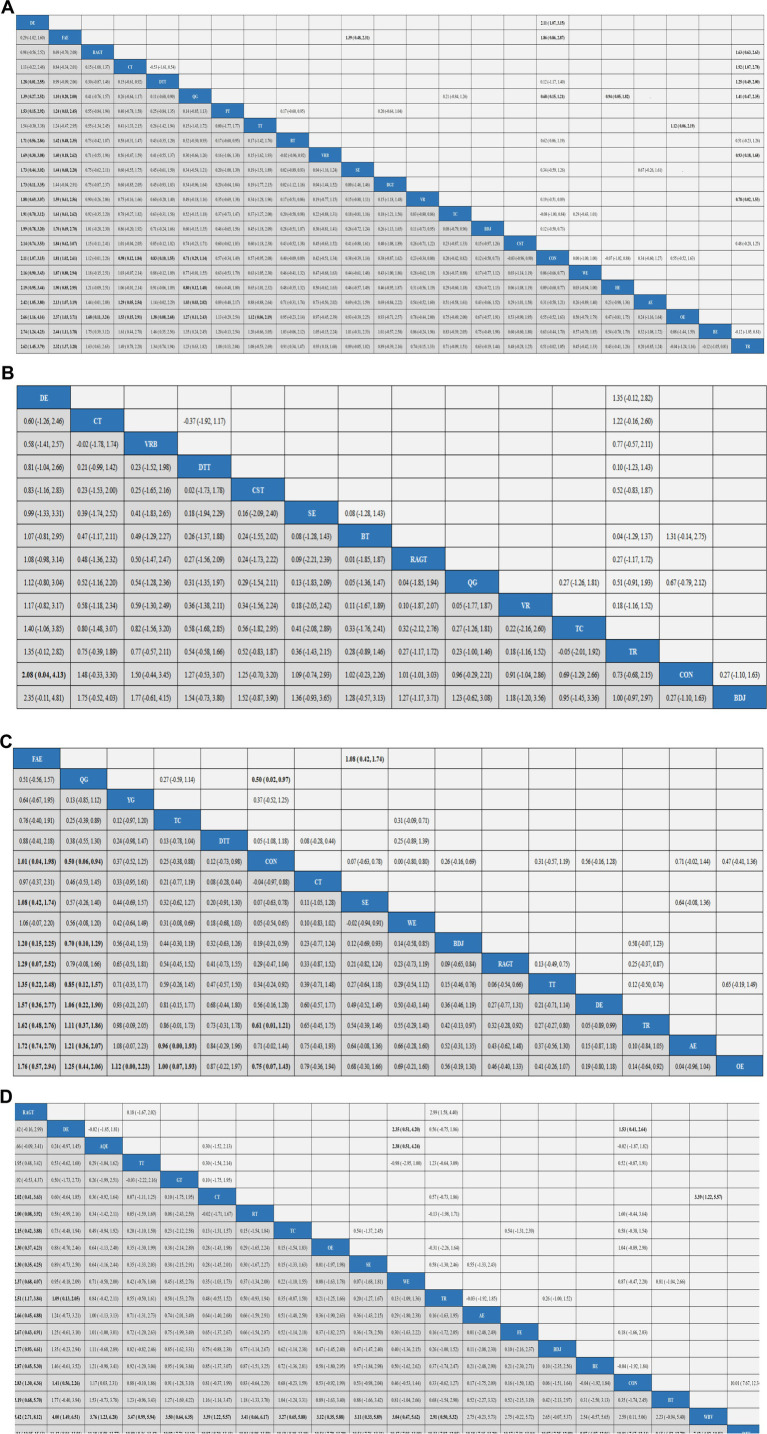
Results analysis league chart. Gait velocity **(A)**, Step length **(B)**, Gait velocity **(C)**, and 6MWT **(D)**. The data are the mean difference and 95% confidence interval of continuous data. Aerobic exercise (AE), Aquatic Exercise (AQE), Whole body vibration training (WBV), Virtual reality (VR), Treadmill training (TT), Resistance training (RT), Tai Chi (TC), Power Training (PT), Biofeedback Balance and Gait Training (BGT), Control group (CON), Walking exercise (WE), Dance exercise (DE), Balance training (BT), Game training (GT), Baduanjin (BDJ), Home exercise (HE), Yoga (YG), Boxing exercise (BE), Robotic Assisted Gait Training (RAGT), Combined therapy (CT), Traditional Rehabilitation (TR), Dual task training (DTT), Stretch exercise (SE), Five animal exercises (FAE), Other exercise (OE), Fitness exercise (FE), Qigong (QG), Virtual reality balance training (VRB), Core strength training (CST).

A total of 33 studies evaluated Gait velocity, involving 1,574 participants. We included the following 23 exercise measures in our network meta-analysis (Figure 3A):

Aerobic exercise (AE), Virtual reality (VR), Treadmill training (TT), Tai Chi (TC), Power Training (PT), Biofeedback Balance and Gait Training (BGT), Control group (CON), Walking exercise (WE), Dance exercise (DE), Balance training (BT), Baduanjin (BDJ), Home exercise (HE), Boxing exercise (BE), Robotic Assisted Gait Training (RAGT), Combined therapy (CT), Traditional Rehabilitation (TR), Dual task training (DTT), Stretch exercise (SE), Five animal exercises (FAE), Other exercise (OE), Qigong (QG), Virtual reality balance training (VRB), Core strength training (CST). Our results show that DE is higher than CON (SMD, 2.11; 95% CI, 1.07 to 3.15), WE (SMD, 2.16; 95% CI 0.90 to 3.43), HE (SMD, 2.19; 95% CI 0.95 to 3.44), AE (SMD, 2.42; 95% CI 1.05 to 3.80), OE (SMD, 2.66; 95% CI 1.16 to 4.16), BE (SMD, 2.74; 95% CI 1.24 to 4.23), TR (SMD2.62; 95% CI 1.45 to 3.79) to better improve Gait velocity in patients with Parkinson’s disease (Figure 4A). In addition, we performed the Egger’s test to assess publication bias (*p* = 0.325) (Appendix 5.1). The included studies did not show publication bias. Heterogeneity and inconsistencies in the mesh meta-analysis were also evaluated (Appendix 6). The most direct comparisons are CON VS QG (Appendix 7.1).

### Step length

A total of 15 studies evaluated GUT, involving 802 participants. We included the following 14 exercise measures in the network meta-analysis (Figure 3B): Virtual reality (VR), Tai Chi (TC), Control group (CON), Dance exercise (DE), Balance training (BT), Baduanjin (BDJ), Robotic Assisted Gait Training (RAGT), Combined therapy (CT), Traditional Rehabilitation (TR), Dual task training (DTT), Stretch exercise (SE), Qigong (QG), Virtual reality balance training (VRB), Core strength training (CST). The results show that DE is superior to CON (SMD, 2.08; 95% CI 0.04 to 4.13) (Figure 4B). In addition, we performed the Egger test to assess publication bias (*p* = 0.239) (Appendix 5.2). The included studies did not show publication bias. We also evaluated the heterogeneity and inconsistencies of the mesh meta-analysis (Appendix 6). We made a direct comparison of exercise interventions (Appendix 7.2).

### Stride length

A total of 19 studies evaluated, involving 741 participants. We included the following 16 exercise measures in the network meta-analysis (Figure 3C): Aerobic exercise (AE), Treadmill training (TT), Tai Chi (TC), Control group (CON), Walking exercise (WE), Dance exercise (DE), Baduanjin (BDJ), Yoga (YG), Robotic Assisted Gait Training (RAGT), Combined therapy (CT), Traditional Rehabilitation (TR), Dual task training (DTT), Stretch exercise (SE), Five animal exercises (FAE), Other exercise (OE), Qigong (QG). The results show that: FAE is superior to CON (SMD, 1.01; 95% CI 0.04 to 1.98), SE (SMD, 1.08; 95% CI 0.42, 1.74), BDJ (SMD, 1.20; 95% CI 0.15 to 2.25), RAGT (SMD, 1.29; 95% CI 0.07 to 2.52), TT (SMD, 1.35; 95% CI 0.22 to 2.48), DE (SMD, 1.57; 95% CI 0.36 to 2.77), TR (SMD, 1.62; 95% CI 0.48 to 2.76), AE (SMD, 1.72; 95% CI 0.74 to 2.70), OE (SMD, 1.76, 95% CI 0.57 to 2.94) in improving Gait velocity (Figure 4C). In addition, we assessed publication bias using the Egger test (*p* = 0.659) (Appendix 5.3). The included studies did not show publication bias. We also evaluated heterogeneity and inconsistencies in the mesh meta-analysis (Appendix 6). We made a direct comparison of exercise interventions (Appendix 7.3). The most direct comparisons are CON vs. QG and BDJ vs. CON.

### 6MWT

A total of 35 studies evaluated, involving 1,616 participants. We included the following 20 exercise measures in the network metaanalysis (Figure 3D): Aerobic exercise (AE), Aquatic Exercise (AQE), Whole body vibration training (WBV), Treadmill training (TT), Resistance training (RT), Tai Chi (TC), Control group (CON), Walking exercise (WE), Dance exercise (DE), Balance training (BT), Game training (GT), Baduanjin (BDJ), Boxing exercise (BE), Robotic Assisted Gait Training (RAGT), Combined therapy (CT), Traditional Rehabilitation (TR), Dual task training (DTT), Stretch exercise (SE), Other exercise (OE), Fitness exercise (FE). The results show that: RAGT is superior to CT (MD, 2.02; 95% CI 0.41 to 3.63), RT (MD, 2.00; 95% CI 0.08, 3.92), TC (MD, 2.15; 95% CI 0.42, 3.88), OE (MD, 2.30; 95% CI 0.37 to 4.23), SE (MD, 2.30; 95% CI 0.35 to 4.25), WE (MD 2.37; 95% CI 0.68 to 4.07), TR (MD, 2.51; 95% CI 1.17 to 3.84), AE (MD2.66; 95% CI 0.45 to 4.88), FE (MD, 2.67; 95% CI 0.43 to 4.91), BDJ (MD, 2.77; 95% CI 0.93 to 4.61), BE (MD, 2.87; 95% CI 0.45 to 5.30), CON (MD, 2.83; 95% CI 1.30 to 4.36), BT (MD, 3.19; 95% CI 0.68 to 5.70), WBV (MD, 5.42; 95% CI 2.71 to 8.21), DTT (MD, 12.84; 95% CI 10.05 to 15.63) in improving 6MWT (Figure 4D). In addition, we assessed publication bias using the Egger test (*p* = 0.688) (Appendix 5.3). The included studies did not show publication bias. We also evaluated heterogeneity and inconsistencies in the mesh meta-analysis (Appendix 6). We made a direct comparison of exercise interventions (Appendix 7.3). The most direct comparisons are CON VS DE.

## Discussion

Gait disorder is one of the common manifestations of motor symptoms in PD patients, which often leads to loss of motor ability and increased mortality. It is one of the essential reasons for the decline in quality of life in PD patients. Therefore, it is of great significance to improve the movement mode of PD patients with gait disorders clearly. A total of 3,114 PD patients were included in our study, and 29 exercise methods were explored to enhance the improvement of gait disorders in PD patients.

Gait velocity and step length are frequently utilized as indicators to monitor the progression of gait disorders and treatment efficacy in patients with PD (Chien et al., 2006). Our study revealed that DE demonstrated superior efficacy compared to the Control group (CON), Walking exercise (WE), Home exercise (HE), Aerobic exercise (AE), Other exercise (OE), Boxing exercise (BE), and Traditional Rehabilitation (TR) in enhancing gait velocity among PD patients. Dance, as an art form, integrates aesthetic imagery, musicality, and goal-setting. It typically begins with a gradual warm-up, potentially improving strength, flexibility, and coordination. Furthermore, improvisation, co-creation, and aesthetic interpretation stimulate participants’ creativity and imagination. The music incorporated in dance provides essential auditory cues for movement through rhythmic and speed variations (Batson et al., 2016). Tactile feedback can also facilitate movement. For instance, when partnered with a submissive individual, rhythmic auditory cues and attention strategies prove more advantageous in improving walking speed during dual tasks (Baker et al., 2007; Westheimer, 2008). Research indicates that DE can mitigate progressive neuronal axon degeneration, promote dendritic formation of new synapses, establish novel neural connections, activate or create new neural pathways, enhance the brain’s regulatory role on limbs, improve joint flexibility, and alleviate movement disorders (Huang and He, 2016). Ashoori et al. (2015) suggested that basal ganglia lesions in PD patients may be associated with reduced internal rhythm, leading to impaired motor initiation and rhythm control. Basal ganglia activity increases during dance, particularly in the core-shell region (Brown et al., 2006). This suggests that dance may beneficially stimulate the basal nucleus in PD patients, enabling them to execute relatively complex movements and enhance their motor abilities. Our meta-analysis demonstrated that exercise interventions can improve step length in PD patients. Interestingly, our study only found DE to be superior to CON in improving step length in PD patients. This implies that most intervention studies in this meta-analysis had negligible or statistically insignificant effects on the step length parameter. Consequently, we recommend that future studies increase their focus on step length in PD research. In addition, our study also found that DE was better than CON in improving step length, but the results may be unstable. Considering that we may have many dances (tango, waltz, samba, Irish dance, ballroom dance, etc.), we suggest a detailed division of dance types for future research. Exploring which kind of dance improves stride length in patients with Parkinson’s disease.

Our results indicate that FAE is superior to Control group (CON), Stretch exercise (SE), Baduanjin (BDJ), Robotic Assisted Gait Training (RAGT), Treadmill training (TT), Dance exercise (DE), Traditional Rehabilitation (TR), Aerobic exercise (AE), and Other exercise (OE) in improving Gait velocity. FAE, a traditional Chinese exercise, primarily emulates the movements of tigers, deer, bears, apes, and birds. This practice effectively stretches muscles and joints, engages various muscle groups, and involves alternating the body’s center of gravity from front to back and left to right, thereby improving movement ability, balance, and coordination. Patients with PD typically exhibit a stooped posture, reduced arm swing, decreased lower limb motion range, slower stride speed, shorter stride length, reduced ground clearance, and prolonged double support period, increasing their susceptibility to falls (Cc and Rc, 2018; Wassom et al., 2015). FAE’s efficacy in improving stride length for PD patients may be attributed to its emphasis on forward stride movements. The exercise requires patients to lift their hips while stepping forward, lunge, swing their arms, and shift their center of gravity between stride types, effectively enhancing their ability to take longer strides and mitigating stride ability decline (Xiaofei and Jinggang, 2018). Given its distinct cultural characteristics, promoting FAE warrants consideration. Strategies for promotion could include organizing basic FAE courses, offering accessible introductory sessions, and providing easily comprehensible multi-language or online courses through digital platforms.

6MWT is a 6-min walking test for patients, which can well reflect the walking endurance and cardiopulmonary function level of patients. Our NMA found that Robotic Assisted Gait Training (RAGT) was superior to Combined therapy (CT), Tai Chi (TC), Other exercise (OE), Stretch exercise (SE), Walking exercise (WE), Traditional Rehabilitation (TR), Aerobic exercise (AE), Fitness exercise (FE), Baduanjin (BDJ), Boxing exercise (BE), Control group (CON), Balance training (BT), Whole body vibration training (WBV), Dual task training (DTT) in improving 6MWT in PD patients. RAGT intervention is grounded in the theory of central nervous system plasticity and functional reorganization. Repetitive, purposeful weight-bearing walking training can enhance balance, facilitating gait automation and improved step speed (Aprile et al., 2019; Toole et al., 2005). Janssen’s study found that enhancing patients’ attention through sensory stimulation increased their walking speed in a straight line (Janssen et al., 2017). RAGT provides diverse sensory stimulation and continuous treatment (Mehrholz et al., 2017), promoting comprehensive motor function recovery and increasing motor ability in PD patients. Furthermore, external rhythm can compensate for defective internal rhythm in the basal ganglia, serving as proprioceptive cues (Nieuwboer et al., 2009), This allows for earlier, more targeted balance training, improving balance and subsequently increasing PD patients’ motor ability (Capecci et al., 2019). Ongoing robot research optimization, exemplified by the new Lokomat robot integrating weight reduction, platform running, and gait correction, enhances timely feedback and evaluation. This enables patients to simulate normal walking under weight reduction conditions (Zhao et al., 2024). Suspension weight reduction maintains patients’ upright posture for balance and reduces walking effort. The WalkBotS robot, supplemented with virtual reality games, achieves direct human-environment interaction. Virtual reality technology provides visual stimulation, better approximating real-life conditions, improving training engagement, and enhancing patient compliance (You et al., 2022). Future research will further explore the effectiveness of this robot-assisted gait training.

### Strengths and limitations

This study represents the most comprehensive and systematic comparative meta-analysis of exercise effects on gait in individuals with PD. With a substantial sample size of 68 studies and 3,114 patients, encompassing 29 exercise interventions, it provides direct and indirect comparisons to offer new, comprehensive, evidence-based recommendations. The study holds significant clinical relevance, demonstrating that DE, FAE, and RAGT can markedly improve gait in PD patients. However, it is important to acknowledge potential limitations of network meta-analysis, such as reliance on indirect comparisons and variability in comparator groups. Notably, exercise emerges as an effective non-pharmacological intervention for PD management. Despite these findings, caution is warranted in interpreting the results due to relatively small sample sizes for each intervention, which may influence outcomes. Gait problems in PD patients worsen with the progression of the disease, and patients have significant individual differences in gait performance (Zhang et al., 2021; Macht et al., 2007). Future studies can focus specifically on each stage of the disease to further reveal the impact of exercise intervention in different disease stages on gait in PD patients. Additionally, natural polyphenols may alleviate bradykinesia, enhance balance and coordination, and reduce turn time in PD patients, possibly due to their anti-striatal oxidative damage properties, inhibition of microglial activation and inflammatory factor secretion, and enhancement of neurotrophic factor expression (Kujawska and Jodynis-Liebert, 2018). Therefore, while focusing on movement interventions for PD patients, it is crucial to consider other factors influencing gait in this population. Previous research has highlighted exercise intensity as a crucial factor influencing exercise effectiveness (Borde et al., 2015; Milanović et al., 2015). However, different exercise modalities used different intensity criteria and intervention duration, which are significant limitations. Some studies included in this analysis failed to report exercise intensity and intervention duration. Future studies should prioritize detailed reporting of exercise intensity and intervention duration and a comparative analysis of gait improvement in PD patients at different intensity levels and intervention times. This approach will help determine the optimal exercise intensity to enhance gait function in PD patients.

## Conclusion

Our study revealed that DE demonstrated superior efficacy in improving gait speed and step length among PD patients compared to other exercise modalities. Furthermore, FAE and RAGT exhibited greater effectiveness in enhancing step length and 6MWT performance, respectively, in individuals with PD. Overall, exercise interventions show significant benefits in improving gait parameters in PD patients. While exercise demonstrates a substantial impact on ameliorating PD symptoms, further investigation is needed to elucidate the underlying molecular mechanisms and neural circuit connections and regulation. Additionally, our quality evaluation highlighted that many studies lacked blinding procedures and randomized grouping, resulting in generally low certainty of evidence. Consequently, we recommend stricter quality control measures in future research, along with increased sample sizes, to further validate the findings of this study. While our study identifies the best form of exercise, safety is a critical consideration for people with Parkinson’s when choosing a form of exercise. To ensure safety, the condition of patients with Parkinson’s disease can be comprehensively considered when choosing exercise methods.

## Data Availability

The original contributions presented in the study are included in the article/Supplementary material, further inquiries can be directed to the corresponding author.

## Glossary

AE: Aerobic exercise
AQE: Aquatic exercise
WBV: Whole body vibration training
VR: Virtual reality
TT: Treadmill training
RT: Resistance training
TC: Tai Chi
PT: Power training
BGT: Biofeedback balance and gait training
CON: Control group
WE: Walking exercise
DE: Dance exercise
BT: Balance training
GT: Game training
BDJ: Baduanjin
HE: Home exercise
YG: Yoga
BE: Boxing exercise
RAGT: Robotic assisted gait training
CT: Combined therapy
TR: Traditional rehabilitation
DTT: Dual task training
SE: Stretch exercise
FAE: Five animal exercises
OE: Other exercise
FE: Fitness exercise
QG: Qigong
VRB: Virtual reality balance training
CST: Core strength training
