# Supplementary material for: Effects of different exercises on improving gait performance in patients with Parkinson’s disease: a systematic review and network meta-analysis
Source: Front Aging Neurosci. 2025 Feb 26;17:1496112. doi: 10.3389/fnagi.2025.1496112 (PMC11897016; doi:10.3389/fnagi.2025.1496112)
Supplement: Supplementary file 1 [file Data_Sheet_1.zip › Supplementary Material/Appendix 1-Search strategy.DOCX]

**Pubmed:**

| Number | Search terms | Results |
| --- | --- | --- |
| **#1** | ((((((((((Parkinson Disease[MeSH Terms]) OR (Idiopathic Parkinson's Disease[Title/Abstract])) OR (Lewy Body Parkinson's Disease[Title/Abstract])) OR (Parkinson's Disease, Idiopathic[Title/Abstract])) OR (Parkinson's Disease, Lewy Body[Title/Abstract])) OR (Paralysis Agitans[Title/Abstract])) OR (Parkinson's Disease[Title/Abstract])) OR (Idiopathic Parkinson Disease[Title/Abstract])) OR (Lewy Body Parkinson Disease[Title/Abstract])) OR (Primary Parkinsonism Parkinsonism, Primary[Title/Abstract])) OR (Parkinson Disease, Idiopathic[Title/Abstract]) | 136,805 |
| **#2** | ((((((((((((((((((((((((((((((((((((((((((((Exercises[Title/Abstract] OR Exercise, Physical[Title/Abstract]) OR (Exercises, Physical[Title/Abstract])) OR (Physical Exercise[Title/Abstract])) OR (Physical Exercises[Title/Abstract])) OR (Physical Activity[Title/Abstract])) OR (Activities, Physical[Title/Abstract])) OR (Activity, Physical[Title/Abstract])) OR (Physical Activities[Title/Abstract])) OR (Exercise, Aerobic[Title/Abstract])) OR (Aerobic Exercise[Title/Abstract])) OR (Aerobic Exercises[Title/Abstract])) OR (Exercises, Aerobic[Title/Abstract])) OR (Exercise, Isometric[Title/Abstract])) OR (Exercises, Isometric[Title/Abstract])) OR (Isometric Exercises[Title/Abstract])) OR (Isometric Exercise[Title/Abstract])) OR (Acute Exercise[Title/Abstract])) OR (Acute Exercises[Title/Abstract])) OR (Realities, Instructional Virtual[Title/Abstract])) OR (Reality, Instructional Virtual[Title/Abstract])) OR (Virtual Realities, Instructional[Title/Abstract])) OR (Treadmill Training[Title/Abstract])) OR (Resistance Training[Title/Abstract])) OR (Tai Chi[Title/Abstract])) OR (Robotic Assisted Gait Training[Title/Abstract])) OR (Power Training[Title/Abstract])) OR (Balance[Title/Abstract] AND Gait Training[Title/Abstract])) OR (Walking[Title/Abstract])) OR (Dance[Title/Abstract])) OR (Balance[Title/Abstract])) OR (Game training[Title/Abstract])) OR (Baduanjin[Title/Abstract])) OR (Pilates exercise[Title/Abstract])) OR (Home exercise[Title/Abstract])) OR (Yoga[Title/Abstract])) OR (Cycling exercise[Title/Abstract])) OR (Boxing exercise[Title/Abstract])) OR (Robotic Training[Title/Abstract])) OR (Core strength training[Title/Abstract])) OR (Dual task training[Title/Abstract])) OR (Stretch exercise[Title/Abstract])) OR (Fitness exercise[Title/Abstract])) OR (Training, Resistance[Title/Abstract])) OR (Strength Training[Title/Abstract])) OR (Training, Strength[Title/Abstract]) | 638,079 |
| **#3** | #1 AND #2 | 7022 |
| **#4** | (((stride length [Title/Abstract]) OR (stride cadence [Title/Abstract])) OR (6 Minutes Walking Test [Title/Abstract])) OR (6MWT[Title/Abstract]) | 9681 |
| **#5** | #3 AND #4 | 500 |
