# Supplementary material for: Effects of different exercises on improving gait performance in patients with Parkinson’s disease: a systematic review and network meta-analysis
Source: Front Aging Neurosci. 2025 Feb 26;17:1496112. doi: 10.3389/fnagi.2025.1496112 (PMC11897016; doi:10.3389/fnagi.2025.1496112)
Supplement: Supplementary file 1 [file Data_Sheet_1.zip › Supplementary Material/Appendix 3-Included literature.docx]

| **Author** | **Publish**  **Year** | **Country** | **measure** | **Age**  **(Mean ± SD)** | **Number**  **(male/** **female)** | **duration of diagnosis**  **(year/month)** | **Intervention**  **time** | **Intervention**  **frequency** | **Outcomes** |
| --- | --- | --- | --- | --- | --- | --- | --- | --- | --- |
| Natale^[1]^ | 2017 | Italy | DT/TR | 66.0±9.15/  70.0±3.16 | 11/16 | 6.0 ±2.07/  6.33± 2.25 | 10 weeks | 2 times a week | ④ |
| Choi^[2]^ | 2013 | Korea | TC/CON | 60.81±7.6/ 65.54±6.8 | NA | 5.2±2.7 | 12 weeks | 3 times a week | ④ |
| Ferraz^[3]^ | 2018 | Brazil | CT/GT | 69±5 | 37/25 | 6±4 | 8 weeks | 3 times a week | ④ |
| Clerici^[4]^ | 2019 | Italy | AQE/CT | 67±9 | 39/13 | NA | 4 weeks | 5 times in five days | ④ |
| Xiao^[5]^ | 2016 | China | BDJ/TR | 67.8±9.4 | NA | NA | 24 weeks | 4 times a week | ④ |
| Conradsson^[6]^ | 2015 | Sweden | BT/SC | 72.9±6.0/  73.6±5.3 | 51/40 | 6.0±5.1/  5.6±5.0 | 10 weeks | NA | ①② |
| Geroin^[7]^ | 2018 | Italy | CT/DTT | 66.05±9.30/ 65.80±9.19 | 88/33 | NA | 6 weeks | 2 times a week | ③ |
| Ribas^[8]^ | 2017 | Brazil | GT/TR | 61.70±6.83/  60.20±11.29 | 8/12 | 6.5±4/  7±2.79 | 12 weeks | 2 times a week | ④ |
| Bang^[9]^ | 2016 | Korea | WE/TT | 58.30±7.71/ 60.60±6.74 | 9/11 | 18.10±6.77/  17.98±3.28 | 4 weeks | 5 times a week | ④ |
| Hackney ^[10]^ | 2009 | America | DE/CON | 68.2±1.4/  66.5±2.8 | 34/14 | NA | 10 weeks | 2 times a week | ④ |
| Hackney^[11]^ | 2007 | America | TC/CON | 60.81±7.6/  65.54±6.8 | NA | 5.2±2.7/  5.2±2.7 | 12 weeks | Once a week | ④③ |
| Shulman^[12]^ | 2013 | America | TT/CT | 66.1±9.7/ 65.3±11.3 | 50/17 | 5.9±3.9/  6.3±4.0 | NA | NA | ④ |
| Solla^[13]^ | 2018 | Italy | DT/TR | 67.8±5.9/ 67.1±6.3 | 13/20 | 4.4 4.5/  5±2.9 | 12 weeks | 2 times a week | ④ |
| Schilling^[14]^ | 2010 | USA | TR/SC | 61.3±8.6 57.0±7.1 | 11/7 | NA | 8 weeks | NA | ④ |
| Picelli^[15]^ | 2013 | Italy | RAGT/TT/TR | 68.50±10.10  68.80±7.72/  67.55±7.08 | 23/37 | 6.52±5.30/  6.99±6.17/  6.79±6.30 | 4 weeks | 3 times a week | ③④ |
| Canning^[16]^ | 2012 | Australia | TT/SC | 60.7±5.9/  62.9±9.9 | 11/9 | 6.1±4.0/  5.2±4.1 | 6 weeks | 4 times a week | ③④ |
| Arfa^[17]^ | 2009 | Iran | TT/CON | 60.63±9.36/  61.55±8.57 | 15/5 | 8.89±5.14)/  8.50±6.34 | 10 weeks | 2 times a week | ④ |
| Xie^[18]^ | 2014 | China | DE/TR | 56. 63±8. 16 57. 80±8. 14 | 56/46 | 3. 57±1. 08/  3. 40±1. 31 | 8 weeks | 3 times a week | ④ |
| Haas^[19]^ | 2024 | Brazil | DE/AQE/WE | 71,61±8,89/ 66.76±8.97/  67.87±11.20 | 35/33 | 5.61±5.09/  8.00±4.65/  7.03±5.07 | 12 weeks | 2 times a week | ④ |
| Khalil^[20]^ | 2017 | Jordan | CT/TR | 58.4±13.5/  60.7±15.4 | 19/11 | 8.0±6.4/  7. 5±4.0 | 8 weeks | Once a week | ④ |
| Dibble ^[21]^ | 2015 | USA | RT/TR | 66.00±14.78/  70.71±9.19 | 25/17 | 8.00±4.48/  5.70±4.23 | 12 weeks | NA | ④ |
| Demonceau^[22]^ | 2017 | belgium | SE/AE/TR | 67±10/65±8 /63.3±6 | 30/15 | 5/7/5 | 12 weeks | 2-3 times a week | ①③④ |
| Zeng^[23]^ | 2020 | China | WBE/CT | 63.27±4.54/ 62.36±3.10 | 18/15 | 6.81±1.60/  6.18±1.32 | 6 weeks | 5 times a week | ④ |
| Cui^[24]^ | 2022 | China | OE/TR | 63.67±5.45/ 65.58±5.38 | 13/11 | 4.83±2.69/  5.75±2.00 | 12 weeks | 2 times a week | ④ |
| Sun^[25]^ | 2022 | China | DTT/TR | 62.9± 4.7/  63.1±4.6 | 49/37 | 5.35±0.84/  5.41±0.89 | 24 weeks | 2 times a week | ①②④ |
| Peng^[26]^ | 2023 | China | WE/BT | 78.14±10.06/  74.00±9.26 | 23/20 | 8.25±3.69/  8.02±3.62 | 12 weeks | 3 times a week | ④ |
| Shi^[27]^ | 2021 | China | CT/TR | 67.89±4.63/  67.48±4.52 | 68.61 | 6.58±1.71/  6.47±1.65 | 8 weeks | 4 times a week | ④ |
| Song^[28]^ | 2020 | China | BDJ/TR | 67.9±3.5/  68.2±3.3 | 68/52 | 2.9±0.5/  2.7±0.3 | 8 weeks | 5 times a week | ④ |
| Ji^[29]^ | 2016 | China | TC/TR | 56.06±11.16/ 59.13±11.22 | 17/15 | 2.09±1.07/  2.28±1.18 | 12 weeks | NA | ①④ |
| Wang^[30]^ | 2017 | China | AQE/TR | 63.93±6.95/ 64.45±6.82 | 38/22 | 4.05±1.55/  4.30±1.66 | 8 weeks | 5 times a week | ④ |
| Liu^[31]^ | 2017 | China | RAGT/TR | 58.7±9.8/  56.5±10.8 | 25/15 | 3.1±1.1/  3.3±0.8 | 10 weeks | 2 times a week | ④ |
| Kunkel^[32]^ | 2017 | UK | DT/TR | 71.3±7.7/ 69.7±6.0 | 25/26 | 4.7±3.5/  7.0±4.9 | 10 weeks | 2 times a week | ④ |
| Combs^[33]^ | 2013 | America | BE/TR | 66.5±28.0/  68.0±31.0 | 21/10 | NA | 12 weeks | NA | ①④ |
| Cugusi^[34]^ | 2015 | Italy | WE/SC | 68.1±8.7/ 66.6±7.3 | 16/4 | 7±2/  7±4 | 12 weeks | 3 times a week | ④ |
| Luan^[35]^ | 2020 | China | TC/SE | 64.08±3.95/ 63.46±4.33 | 13/13 | NA | 16 weeks | 3 times a week | ④ |
| Mak^[36]^ | 2021 | China | WE/CON | 61.9±6.4/ 62.7±7.2 | 44/20 | 5.8±6.0/  5.0±4.0 | 24 weeks | 2-3 times a week | ④ |
| Gao^[37]^ | 2022 | China | TC/ FE/CON | 64±5/65±8/  63±6 | 24/39 | NA | 16 weeks | 3 times a week | ④ |
| Haputhanthirige^[38]^ | 2023 | Australia | DE/CON | 65.24±11.88/  66.50±7.70 | 13/20 | 3.76±2.88/  5.94±3.61 | 12 weeks | 2 times a week | ①② |
| Wan^[39]^ | 2021 | China | QG/CON | 64.95±7.83/  67.03±7.47 | 19/21 | 3.63±1.52/  3.25±1.73 | 12 weeks | 4 times a week | ①③ |
| Li^[40]^ | 2022 | China | QG/CON | 64.72±7.29 | 11/20 | NA | 12 weeks | 5 times a week | ①③ |
| Fisher^[41]^ | 2008 | America | OE/CON | 63.1±11.5/  64.0±14.5 | 19/11 | NA | 8 weeks | NA | ①③ |
| Shen^[42]^ | 2014 | China | BGT/PT | 63.3±8.0/  65.3±8.5 | 25/20 | 2.4±0.5/  2.5±0.5 | 12 weeks | 3 times a week | ① |
| Lei^[43]^ | 2017 | Canada | WE/CON | 61.58±11.7/  68±6.4 | 13/11 | NA | 6 weeks | 3-4 times a week | ①③ |
| Amano^[44]^ | 2013 | USA | TC/QG | 64±13/  68±7 | 12/7 | 7±7/  12±7 | 16 weeks | 2 times a week | ①②③ |
| Wong^[45]^ | 2015 | China | BT/TR | 59.4±9.0/ 62.6±8.9 | 46/32 | 7.1±4.3/  5.6±3.8 | 8 weeks | Once a week | ① |
| Schlick^[46]^ | 2016 | Germany | OE/TT | 71.2±10.9/  68.9±6.8 | 6/14 | 10.4±5.2/  9±3.1 | 5 weeks | 2-3 times a week | ①③ |
| Vasconcellos^[47]^ | 2021 | Brazil | HE/CON | 66.0±6.3/ 65.4±10.1 | 18/10 | 6.1±4.4/  6.0±4.7 | 3 weeks | NA | ① |
| Rosenfeldt ^[48]^ | 2019 | USA | CT/DTT | 65±8/  59±9 | 14/6 | 4/8 | 8 weeks | 3 times a week | ①② |
| Fok^[49]^ | 2010 | Australia | DTT/CON | 66.8±9.0/ 57.7±12.3 | NA | 4.2±2.4/  5.5±3.8 | NA | NA | ①③ |
| Li^[50]^ | 2021 | China | FAE/SE | 67.57±3.95/  70±5.59 | 29/11 | 6.83±4.09/  7.76±4.55 | 12 weeks | 2 times a week | ①③ |
| Chang^[51]^ | 2020 | China | CT/TR | 69±2/67±11 | 76/44 | NA | 11 weeks | Once a day | ① |
| LV^[52]^ | 2021 | China | QG/SC | 65.87±6.13/ 63.25±6.70 | 11/20 | 5.60±1.72/6.13±1.96 | 12 weeks | 5 times a week | ①② |
| Dong^[53]^ | 2022 | China | BDJ/CON | 65.37±7.47 63.07±12.78 | 29/38 | 6.63±2.27/6.67±2.24 | 3 weeks | Once a week | ①②③ |
| Zhi^[54]^ | 2020 | China | BDJ/CON | 56. 2±9. 90 58. 0±4. 98 | 16/14 | 3. 00±2. 05/3. 25±1. 65 | 12 weeks | 6 times a week | ①③ |
| Zong^[55]^ | 2021 | China | QG/TR | NA | NA | NA | 12 weeks | 5 times a week | ①② |
| Zhang^[56]^ | 2019 | China | FAE/CON | 65.06±4.905/ 63.94±5.068 | 13/21 | 2.59±1.58/2.47±1.12 | 48 weeks | 2 times a week | ① |
| Lin^[57]^ | 2021 | China | CT/TR | 66.7±5.3/ 69.5±2.4 | 15/17 | 5.3±0.6/4.2±1.1 | 8 weeks | 5 times a week | ①② |
| Qin^[58]^ | 2019 | China | VR/TR | 65.9±4.9/  66.1±6.2 | 45/34 | 7.9±2.7/8.1±2.3 | 6 weeks | 5 times a week | ①② |
| Yu^[59]^ | 2015 | China | GST/TR | 63.5±8.2/ 65.2±7.4 | 52/29 | 3.9±1.4/4.1±1.3 | 12 weeks | NA | ①② |
| Tang^[60]^ | 2017 | China | PT/BT | 74.48±6.31/ 75.63±5.82 | 49/15 | 10.5/9.3 | 8 weeks | 2 times a week | ①② |
| Guo^[61]^ | 2018 | China | QG/HE | 65.15±3.74/ 63.95±6.68 | 16/24 | NA | 12 weeks | 2 times a week |  |
| Lu^[62]^ | 2024 | China | CT/TR | 66.70±6.73/  66.57±6.82 | 34/26 | 5.93±2.34/  5.23±2.39 | 4 weeks | Once a week | ①② |
| Xiao^[63]^ | 2015 | China | TC/WE | 68.17±2.27/  66.52±2.13 | 67/29 | 5.45±3.61/6.15±2.63 | 24 weeks | 4 times a week | ①③ |
| Yang^[64]^ | 2019 | China | DTT/WE | 65.0 (57.5–75.8) /  66.5 (55.5–76.5) | 12/6 | 5.5 (2.8-10.5)/ 3.0(0.3-10.0) | 4 weeks | 3 times a week | ③ |
| Cheung^[65]^ | 2018 | USA | YG/CON | 63.5±8.5/  65.8±6.6 | NA | 4.8± 2.9 | 12 weeks | 2 times a week | ③ |
| Wu^[66]^ | 2024 | China | BDJ/TR | 66.72±6.00/66.5±5.90 | 23/15 | 64.11±29.81M/ 63.85±27.99 | 4 weeks | 5 times a week | ③ |
| Zhang^[67]^ | 2022 | China | BT/CON | 67.00±6.17/71.89±8. 06 | 19/19 | NA | 4 weeks | 5 times a week | ①② |
| He^[68]^ | 2022 | China | VRB/TR | 64.48±13.4/62.95±14.21 | 54/28 | NA | 4 weeks | 5 times a week | ①② |

1. : Gait velocity (m/s); ②: Step length (m) ; ③: Stride length (m) ; ④: 6MWT(m)

[1] DE NATALE ER, PAULUS KS, AIELLO E, et al. Dance therapy improves motor and cognitive functions in patients with Parkinson's disease. NeuroRehabilitation. 2017;40(1):141-144.

[2] CHOI HJ, GARBER CE, JUN TW, et al. Therapeutic effects of tai chi in patients with Parkinson's disease. ISRN Neurol. 2013;2013:548240.

[3] FERRAZ DD, TRIPPO KV, DUARTE GP, et al. The Effects of Functional Training, Bicycle Exercise, and Exergaming on Walking Capacity of Elderly Patients With Parkinson Disease: A Pilot Randomized Controlled Single-blinded Trial. Arch Phys Med Rehabil. 2018;99(5):826-833.

[4] CLERICI I, MAESTRI R, BONETTI F, et al. Land Plus Aquatic Therapy Versus Land-Based Rehabilitation Alone for the Treatment of Freezing of Gait in Parkinson Disease: A Randomized Controlled Trial. Phys Ther. 2019;99(5):591-600.

[5] XIAO C, ZHUANG Y, KANG Y. Effect of Health Qigong Baduanjin on Fall Prevention in Individuals with Parkinson's Disease. J Am Geriatr Soc. 2016;64(11):e227-e228.

[6] CONRADSSON D, LöFGREN N, NERO H, et al. The Effects of Highly Challenging Balance Training in Elderly With Parkinson's Disease: A Randomized Controlled Trial. Neurorehabil Neural Repair. 2015;29(9):827-836.

[7] GEROIN C, NONNEKES J, DE VRIES NM, et al. Does dual-task training improve spatiotemporal gait parameters in Parkinson's disease? Parkinsonism Relat Disord. 2018;55:86-91.

[8] RIBAS CG, ALVES DA SILVA L, CORRêA MR, et al. Effectiveness of exergaming in improving functional balance, fatigue and quality of life in Parkinson's disease: A pilot randomized controlled trial. Parkinsonism Relat Disord. 2017;38:13-18.

[9] BANG DH, SHIN WS. Effects of an intensive Nordic walking intervention on the balance function and walking ability of individuals with Parkinson's disease: a randomized controlled pilot trial. Aging Clin Exp Res. 2017;29(5):993-999.

[10] HACKNEY ME, EARHART GM. Effects of dance on movement control in Parkinson's disease: a comparison of Argentine tango and American ballroom. J Rehabil Med. 2009;41(6):475-481.

[11] HACKNEY ME, KANTOROVICH S, LEVIN R, et al. Effects of tango on functional mobility in Parkinson's disease: a preliminary study. J Neurol Phys Ther. 2007;31(4):173-179.

[12] SHULMAN LM, KATZEL LI, IVEY FM, et al. Randomized clinical trial of 3 types of physical exercise for patients with Parkinson disease. JAMA Neurol. 2013;70(2):183-190.

[13] SOLLA P, CUGUSI L, BERTOLI M, et al. Sardinian Folk Dance for Individuals with Parkinson's Disease: A Randomized Controlled Pilot Trial. J Altern Complement Med. 2019;25(3):305-316.

[14] SCHILLING BK, PFEIFFER RF, LEDOUX MS, et al. Effects of moderate-volume, high-load lower-body resistance training on strength and function in persons with Parkinson's disease: a pilot study. Parkinsons Dis. 2010;2010:824734.

[15] PICELLI A, MELOTTI C, ORIGANO F, et al. Robot-assisted gait training versus equal intensity treadmill training in patients with mild to moderate Parkinson's disease: a randomized controlled trial. Parkinsonism Relat Disord. 2013;19(6):605-610.

[16] CANNING CG, ALLEN NE, DEAN CM, et al. Home-based treadmill training for individuals with Parkinson's disease: a randomized controlled pilot trial. Clin Rehabil. 2012;26(9):817-826.

[17] ARFA-FATOLLAHKHANI P, SAFAR CHERATI A, HABIBI SAH, et al. Effects of treadmill training on the balance, functional capacity and quality of life in Parkinson's disease: A randomized clinical trial. J Complement Integr Med. 2019;17(1).

[18] DONGQIN X, LING Y, LANYUN Y. Effects of dance training combined with cognitive intervention on cognitive function, motor function and coping style of patients with Parkinson's disease and prognosis analysis. Journal of Navy Medicine. 2024;45(03):323-326.

[19] HAAS AN, DELABARY MDS, PASSOS-MONTEIRO E, et al. The effects of Brazilian dance, deep-water exercise and nordic walking, pre- and post-12 weeks, on functional-motor and non-motor symptoms in trained PwPD. Arch Gerontol Geriatr. 2024;118:105285.

[20] KHALIL H, BUSSE M, QUINN L, et al. A pilot study of a minimally supervised home exercise and walking program for people with Parkinson's disease in Jordan. Neurodegener Dis Manag. 2017;7(1):73-84.

[21] DIBBLE LE, FOREMAN KB, ADDISON O, et al. Exercise and medication effects on persons with Parkinson disease across the domains of disability: a randomized clinical trial. J Neurol Phys Ther. 2015;39(2):85-92.

[22] DEMONCEAU M, MAQUET D, JIDOVTSEFF B, et al. Effects of twelve weeks of aerobic or strength training in addition to standard care in Parkinson's disease: a controlled study. Eur J Phys Rehabil Med. 2017;53(2):184-200.

[23] DUCHUN Z, LIANG T, TONGCAI T, et al. Effects of whole body vibration combined with multiple motor strategy training on motor function and daily living ability of patients with Parkinson's disease. Chinese Journal of Rehabilitation Medicine. 2020;35(12):1486-1488.

[24] BINGKUN C. Study on the effect of simple diabolo rehabilitation exercise on motor ability and depressive psychology of patients with mild Parkinson's disease. 2022.

[25] WEI S. Application effect of dual task training in patients with Parkinson's motor dysfunction. Modern Nurse. 2022;29(12):124-128.

[26] AIJUN P, YINPING X, ZHAOYING P. Effects of body weight support treadmill training on cardiopulmonary endurance, balance ability and quality of life of elderly patients with Parkinson’s disease. Shanghai Pharmaceutical. 2023;44(06):51-54.

[27] XINWEI S, WEILI Y, JUN J. Effects of Baduanjin combined with balance pad training on lower limb motor function and body balance in elderly patients with Parkinson's disease. Practical clinical integration of traditional Chinese and Western medicine. 2021;21(11):56-57.

[28] ZHENGXIN S, ZENGLIN C, MIN W, et al. The value of Baduanjin combined with balance mat training in improving balance function in elderly patients with Parkinson's disease. Chinese Journal of Practical Nursing. 2020;36(2):100-104.

[29] SUGIONG J, ZHIJUAN M, QINGMEI Y. Effectiveness of Tai Chi for Parkinson disease. Chinese Journal of Rehabilitation. 2016;31(01):51-53.

[30] YI⁃ZHAO W, HUA Z, SHI⁃CHUN F, et al. Effect of water⁃based exercise onmotor function, balance function andwalking ability in patients with Parkinson's disease. ChinJ Contemp Neurol Neurosurg. 2017;17(05):346-351.

[31] PING L, MEIYUN C. Eficacy of Lokomat roboticassisted gait training on improving gait function in patients with Parkinson's disease Chinese Journal of Rehabilitation. 2017;32(01):30-32.

[32] KUNKEL D, FITTON C, ROBERTS L, et al. A randomized controlled feasibility trial exploring partnered ballroom dancing for people with Parkinson's disease. Clin Rehabil. 2017;31(10):1340-1350.

[33] COMBS SA, DIEHL MD, CHRZASTOWSKI C, et al. Community-based group exercise for persons with Parkinson disease: a randomized controlled trial. NeuroRehabilitation. 2013;32(1):117-124.

[34] CUGUSI L, SOLLA P, SERPE R, et al. Effects of a Nordic Walking program on motor and non-motor symptoms, functional performance and body composition in patients with Parkinson's disease. NeuroRehabilitation. 2015;37(2):245-254.

[35] XIN L. Effects of Innovative Tai Chi Training on Motor Ability and Quality of Life in Patients with Early Parkinson’s Disease. 2020.

[36] MAK MKY, WONG-YU ISK. Six-Month Community-Based Brisk Walking and Balance Exercise Alleviates Motor Symptoms and Promotes Functions in People with Parkinson's Disease: A Randomized Controlled Trial. J Parkinsons Dis. 2021;11(3):1431-1441.

[37] SUN G. Research on the Effects of Tai Chi on Motor Symptoms of Patients with Parkinson’s Disease and Its Promotion Based on the Internet Platform in Song Gao. 2022.

[38] HAPUTHANTHIRIGE NKH, SULLIVAN K, MOYLE G, et al. Effects of dance on gait and dual-task gait in Parkinson's disease. PLoS One. 2023;18(1):e0280635.

[39] WAN Z, LIU X, YANG H, et al. Effects of Health Qigong Exercises on Physical Function on Patients with Parkinson's Disease. J Multidiscip Healthc. 2021;14:941-950.

[40] LI X, LV C, LIU X, et al. Effects of Health Qigong Exercise on Lower Limb Motor Function in Parkinson's Disease. Front Med (Lausanne). 2021;8:809134.

[41] FISHER BE, WU AD, SALEM GJ, et al. The effect of exercise training in improving motor performance and corticomotor excitability in people with early Parkinson's disease. Arch Phys Med Rehabil. 2008;89(7):1221-1229.

[42] SHEN X, MAK MK. Balance and Gait Training With Augmented Feedback Improves Balance Confidence in People With Parkinson's Disease: A Randomized Controlled Trial. Neurorehabil Neural Repair. 2014;28(6):524-535.

[43] ZHOU L, GOUGEON MA, NANTEL J. Nordic Walking Improves Gait Power Profiles at the Knee Joint in Parkinson's Disease. J Aging Phys Act. 2018;26(1):84-88.

[44] AMANO S, NOCERA JR, VALLABHAJOSULA S, et al. The effect of Tai Chi exercise on gait initiation and gait performance in persons with Parkinson's disease. Parkinsonism Relat Disord. 2013;19(11):955-960.

[45] WONG-YU IS, MAK MK. Multi-dimensional balance training programme improves balance and gait performance in people with Parkinson's disease: A pragmatic randomized controlled trial with 12-month follow-up. Parkinsonism Relat Disord. 2015;21(6):615-621.

[46] SCHLICK C, ERNST A, BöTZEL K, et al. Visual cues combined with treadmill training to improve gait performance in Parkinson's disease: a pilot randomized controlled trial. Clin Rehabil. 2016;30(5):463-471.

[47] VASCONCELLOS LS, SILVA RS, PACHêCO TB, et al. Telerehabilitation-based trunk exercise training for motor symptoms of individuals with Parkinson's disease: A randomized controlled clinical trial. J Telemed Telecare. 2023;29(9):698-706.

[48] ROSENFELDT AB, PENKO AL, STREICHER MC, et al. Improvements in temporal and postural aspects of gait vary following single- and multi-modal training in individuals with Parkinson's disease. Parkinsonism Relat Disord. 2019;64:280-285.

[49] FOK P, FARRELL M, MCMEEKEN J. Prioritizing gait in dual-task conditions in people with Parkinson's. Hum Mov Sci. 2010;29(5):831-842.

[50] ZHENLAN L. Effects of Exercise Intervention on Gait during Different Tasks for Patients with Mild to Moderate Parkinson's disease. 2021.

[51] ZIHAN C. Inflammation and oxidative stress in PD and evaluation theefficacy of intensive training & virtual reality rehabilitationon gait disorder and motor function in PD patients. 2020.

[52] CHUANFANG L. Effects of Health Qigong exercise on lower limb motor functionin patients with Parkinson's disease. 2021.

[53] SS D, HJ Y, DONGQ. Effects of Baduanjin training on gait and balance function of patients with Parkinson's disease. Rehabilitation Medicine. 2022;32(01):18-24+31.

[54] XIAO Z. Effects of Baduanjin on Gait Parameters in Individuals with Parkinson's Disease 2020.

[55] WEIJIE Z, HUI Y, MEILING W, et al. Studyon theEffect ofHealth QigongExerciseIntervention in Gait in Parkinson’sPatients. Journal of Capital Physical Education Institute. 2021;33(03):294-298+315.

[56] GAI Z. Research on the Intervention Effect of Health Qigong Wuqinxi on theWalking and Balance Ability of Patients with Parkinson's Disease. 2019.

[57] ZAILONG L, XIONGWEI F, CHAOWEI Y, et al. Effect of Flexbot lower extremity rehabilitation robot combined with virtual reality training on balance function and walking ability of patients with Parkinson's disease. Zhejiang medical science. 2021;43(04):405-408+413.

[58] LINGZHI Q, WEI L, XIAOJUAN W, et al. Application of virtual reality technology in frozen gait rehabilitation of Parkinson's disease. Chinese Journal of Physical Medicine and Rehabilitation. 2019;41(3):206-209.

[59] MEI Y, LIANTAO L, TONGBAO D, et al. Effect of strengthening core muscle strength training on rehabilitation of Parkinson's disease. Guangdong medicine. 2015;36(01):77-79.

[60] JIE T, YAN Z, BIYING L. Effects of strength and balance training on motor and postural control in patients with Parkinson's disease. Neural Injury And Functional Reconstruction. 2017;12(03):266-268.

[61] YADONG G. Effects of Health Qigong on Motor Function in Patientswith mild to Moderate Parkinson's Disease. 2018.

[62] CHUNXIA L, YOUZHEN Z, XIANRONG M, et al. Clinical effect of Baduanjin exercise combined with G-EO rehabilitation robot in treatment of dyskinesia due to Parkinson’s disease:An analysis of 30 cases. HUNAN JOURNAL OF TRADITIONAL CHINESE MEDICINE. 2024;40(04):18-21.

[63] XIAO CM, ZHUANG YC. Effect of health Baduanjin Qigong for mild to moderate Parkinson's disease. Geriatr Gerontol Int. 2016;16(8):911-919.

[64] YANG YR, CHENG SJ, LEE YJ, et al. Cognitive and motor dual task gait training exerted specific training effects on dual task gait performance in individuals with Parkinson's disease: A randomized controlled pilot study. PLoS One. 2019;14(6):e0218180.

[65] CHEUNG C, BHIMANI R, WYMAN JF, et al. Effects of yoga on oxidative stress, motor function, and non-motor symptoms in Parkinson's disease: a pilot randomized controlled trial. Pilot Feasibility Stud. 2018;4:162.

[66] MAODONG W, ZHENJIE S, QINGLUN S, et al. Effects of Baduanjin on gait parameters and serum nerve growth factor in patients with Parkinson's disease with frozen gait. Chinese Journal of Behavioral Medicine and Brain Sciences. 2024;33(3):212-218.

[67] YING Z, HUI Q, YAJUN Z, et al. The effects of metoba combined with balance function training on balance and walking ability of patients with Parkinson's disease were studied based on gait analysis. Chinese Journal of Gerontology. 2022;42(14):3478-3480.

[68] RONG H, LEI J. Intervention effect of virtual reality balance game combined with progressive rehabilitation nursing in patients with Parkinson's disease. Life science instrument. 2022;20(z1):192-193.
