# Supplementary material for: Effects of different exercises on improving gait performance in patients with Parkinson’s disease: a systematic review and network meta-analysis
Source: Front Aging Neurosci. 2025 Feb 26;17:1496112. doi: 10.3389/fnagi.2025.1496112 (PMC11897016; doi:10.3389/fnagi.2025.1496112)
Supplement: Supplementary file 1 [file Data_Sheet_1.zip › Supplementary Material/Appendix 4-Risk of bias summary.pdf]

	Random sequence generation (selection bias)	Allocation concealment (selection bias)	Binding of participants and personnel (performance bias)	Binding of outcome assessment (detection bias)	Incomplete outcome data (attrition bias)	Selective reporting (reporting bias)	Other bias
Amano 2013	+	−	−	−	+	+	+
Arfa 2009	+	+	−	−	+	+	+
Bang 2016	+	+	+	?	+	+	+
Canning 2012	+	+	?	+	+	+	+
Chang 2020	+	+	−	−	+	+	+
Cheung 2018	+	+	−	−	+	+	+
Choi 2013	+	?	−	−	+	+	+
Clerici 2019	+	+	+	?	+	+	+
Combs 2013	+	+	?	+	+	+	+
Conradsson 2015	+	+	−	−	+	+	+
Cugusi 2015	+	+	−	−	+	+	+
Cui 2022	+	?	−	−	+	+	+
Demonceau 2017	+	+	?	?	+	+	+
Dibble 2015	−	−	+	?	+	+	+
Dong 2022	+	+	−	−	+	+	+
Ferraz 2018	+	+	+	+	+	+	+
Fishe 2008	+	+	−	−	+	+	+
Fok 2010	?	?	−	−	+	+	+
Gao 2022	+	+	−	+	+	+	+
Geroin 2018	+	+	?	+	+	+	+
Guo 2018	+	+	−	−	+	+	+
Haas 2024	+	+	+	+	+	+	+
Hackney 2007	+	+	−	−	+	+	+
Hackney 2009	+	+	?	?	+	+	+
Haputhanthirige 2023	?	?	−	−	+	+	+
He 2022	+	+	−	−	+	+	+
Ji 2016	+	+	−	−	+	+	+
Khali 2017	+	+	?	+	+	+	+
Kunkel 2017	?	+	?	+	+	+	+
Lei 2017	+	+	−	−	+	+	+
Li 2021	+	+	−	−	+	+	+
Li 2022	+	+	−	−	+	+	+
Lin 2021	+	+	−	−	+	+	+
Liu 2017	+	+	−	−	+	+	+
Lu 2024	+	+	−	−	+	+	+
Luan 2020	+	+	−	−	+	+	+
LV 2021	+	−	−	−	+	+	+
Mak 2021	+	+	?	+	+	+	+
Natale 2017	+	?	−	−	+	+	+
Peng 2023	+	+	−	−	+	+	+
Picelli 2013	+	+	−	−	+	+	+
Qin 2019	+	+	−	−	+	+	+
Ribas 2017	+	+	?	?	+	+	+
Rosenfeldt 2019	+	+	−	+	+	+	+
Schilling 2010	+	+	−	−	+	+	+
Schlick 2016	?	?	−	−	+	+	+
Shen 2014	+	+	−	−	+	+	+
Shi 2021	+	+	−	−	+	+	+
Shulman 2013	+	+	?	?	+	+	+
Solla 2018	+	+	?	+	+	+	+
Song 2020	?	?	−	−	+	+	+
Sun 2022	+	+	?	?	+	+	+
Tang 2017	+	+	−	−	+	+	+
Vasconcellos 2021	+	+	?	+	+	+	+
Wan 2021	+	+	−	+	+	+	+
Wang 2017	+	+	−	−	+	+	+
Wong 2015	+	+	−	−	+	+	+
Wu 2024	+	+	−	−	+	+	+
Xiao 2015	+	+	−	−	+	+	+
Xiao 2016	+	+	?	?	+	+	+
Xie 2014	+	+	−	−	+	+	+
Yang 2019	+	+	−	−	+	+	+
Yu 2015	+	+	−	−	+	+	+
Zeng 2020	+	+	−	−	+	+	+
Zhang 2019	+	+	−	−	+	+	+
Zhang 2022	+	+	−	−	+	+	+
Zhi 2020	+	+	−	−	+	+	+
Zong 2021	+	+	−	−	+	+	+
