# Supplementary figures and images for: Effects of different exercises on improving gait performance in patients with Parkinson’s disease: a systematic review and network meta-analysis
Source: Front Aging Neurosci. 2025 Feb 26;17:1496112. doi: 10.3389/fnagi.2025.1496112 (PMC11897016; doi:10.3389/fnagi.2025.1496112)

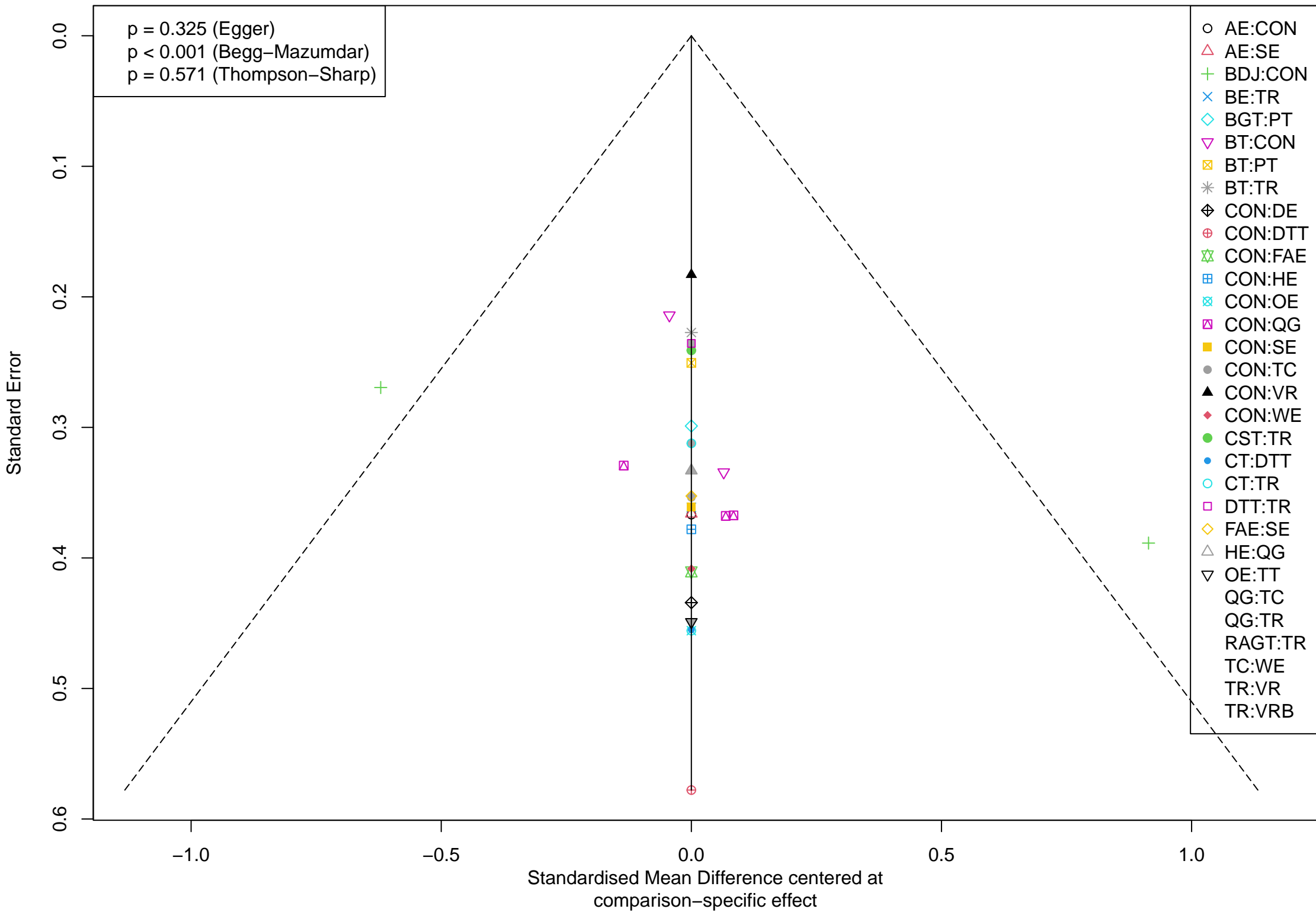

Supplement: Supplementary file 1 [file Data_Sheet_1.zip › Supplementary Material/Appendix 5.1-Funnel polt.pdf]

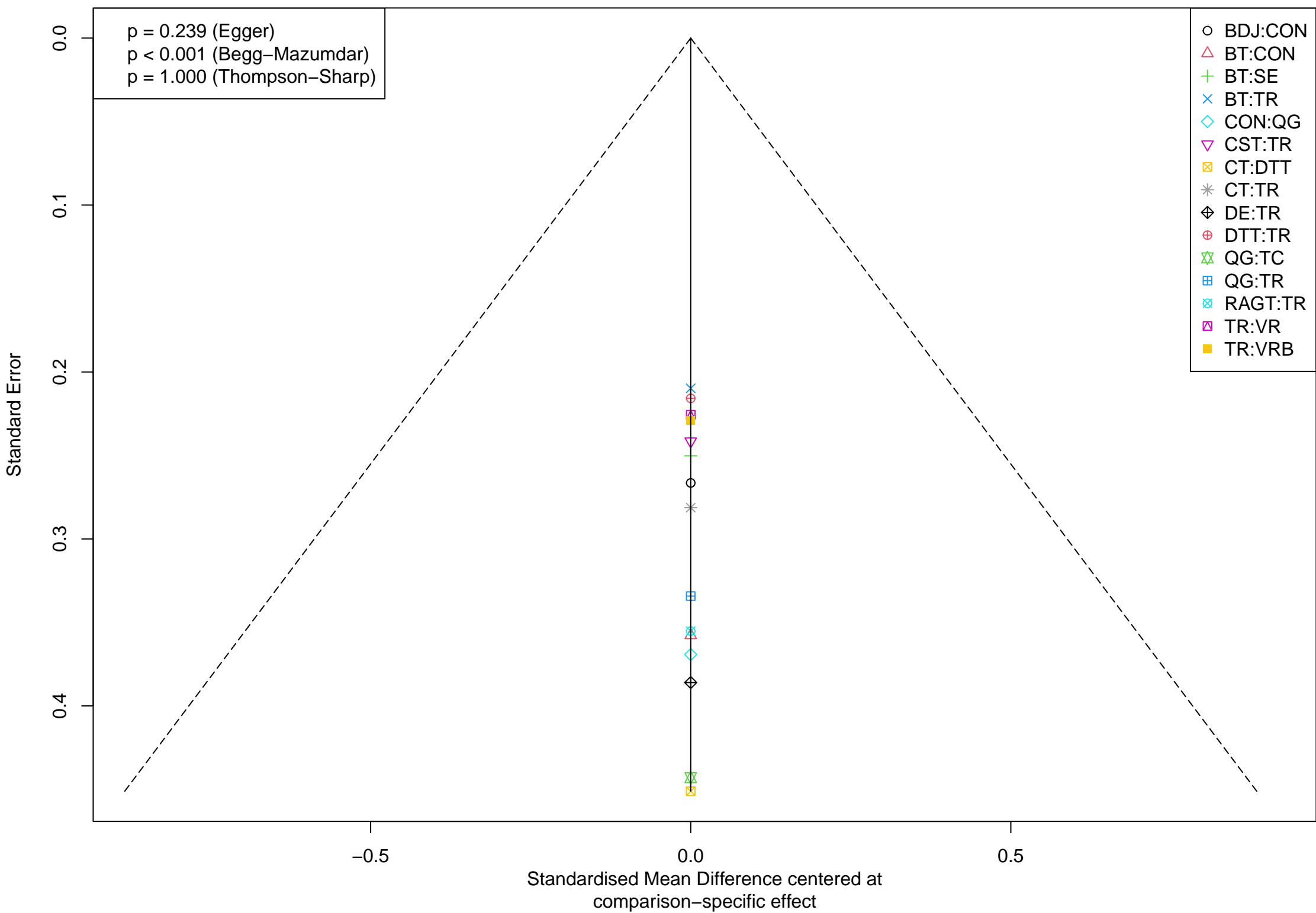

Supplement: Supplementary file 1 [file Data_Sheet_1.zip › Supplementary Material/Appendix 5.2-Funnel polt.pdf]

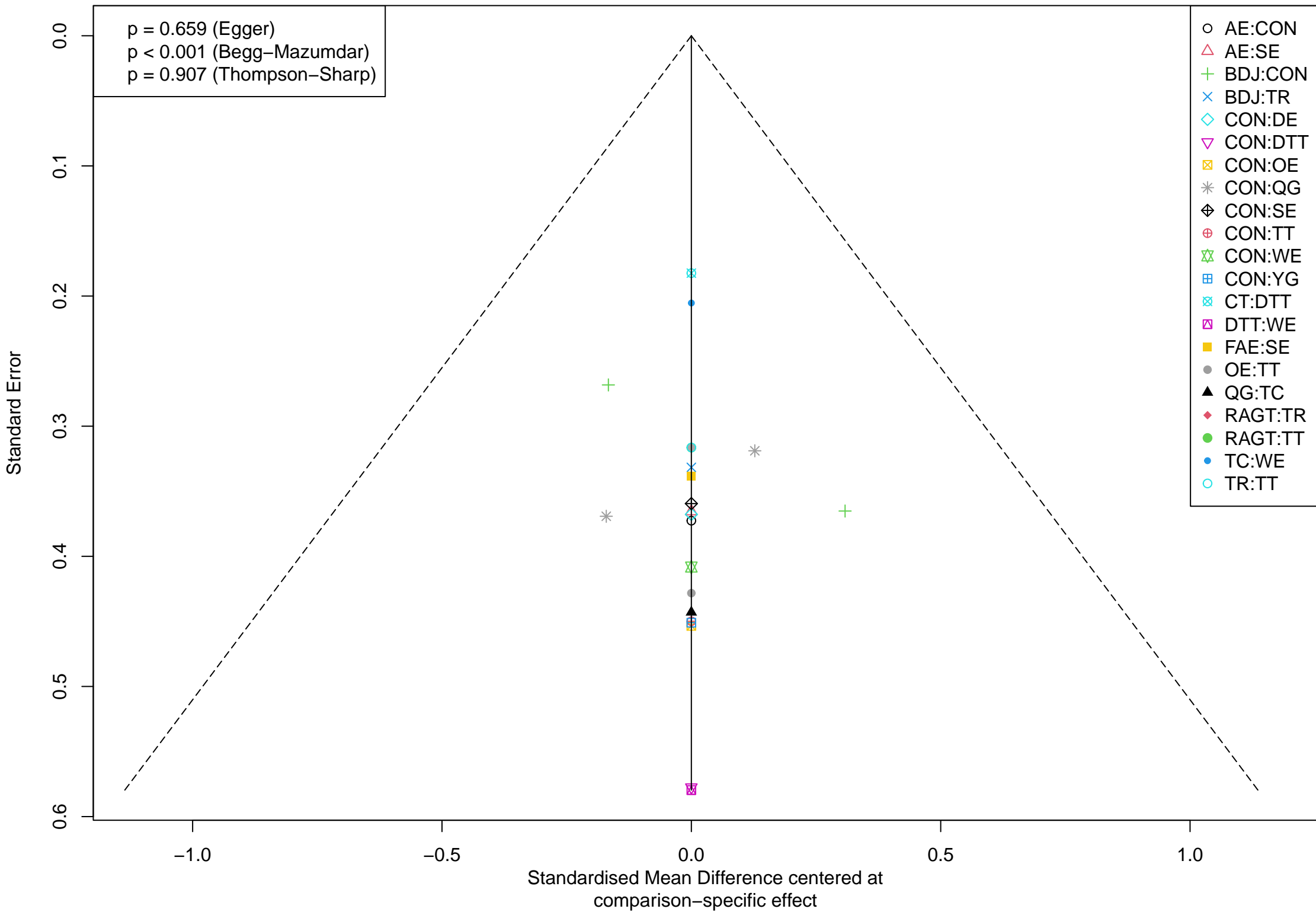

Supplement: Supplementary file 1 [file Data_Sheet_1.zip › Supplementary Material/Appendix 5.3-Funnel polt.pdf]

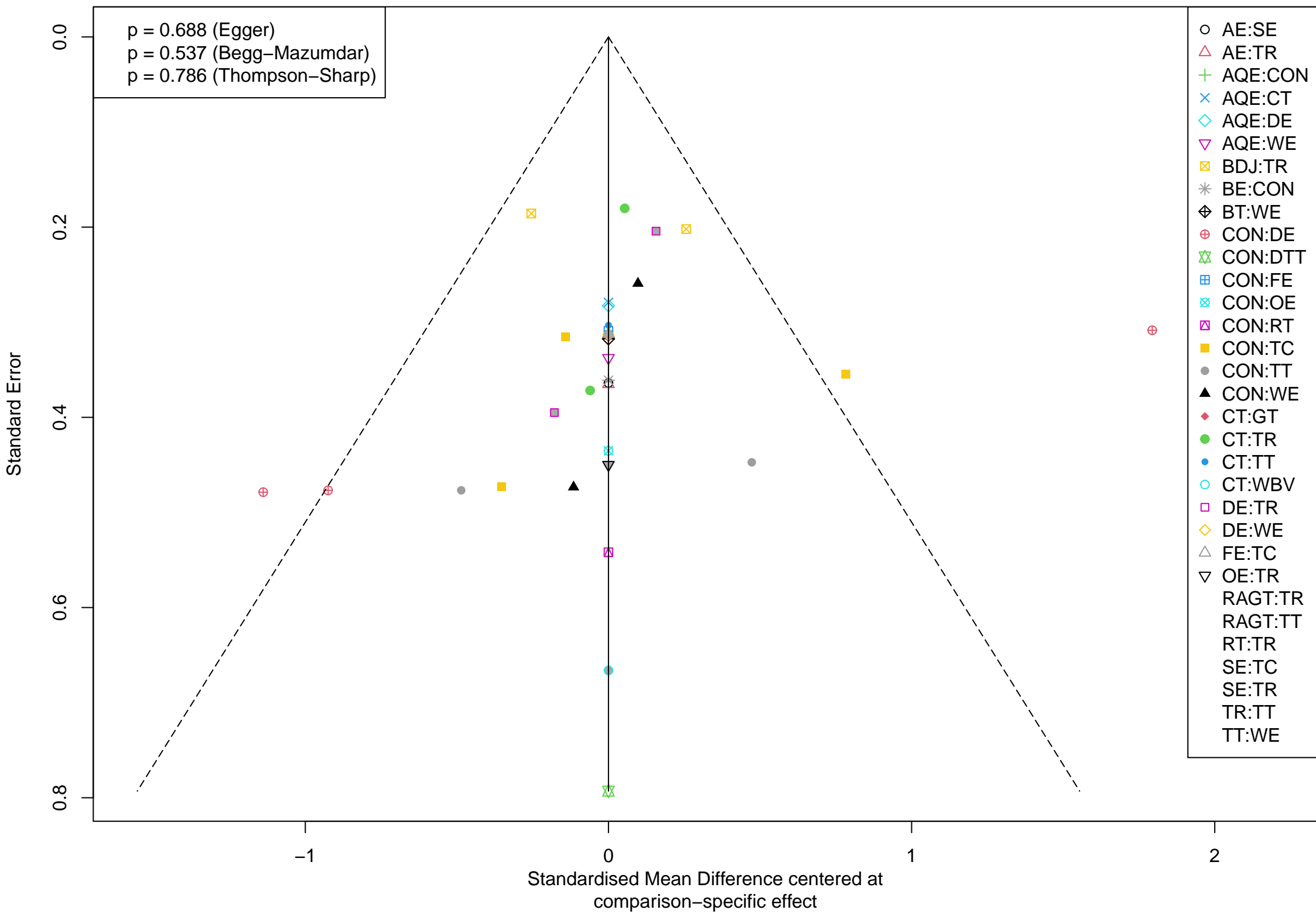

Supplement: Supplementary file 1 [file Data_Sheet_1.zip › Supplementary Material/Appendix 5.4-Funnel polt.pdf]

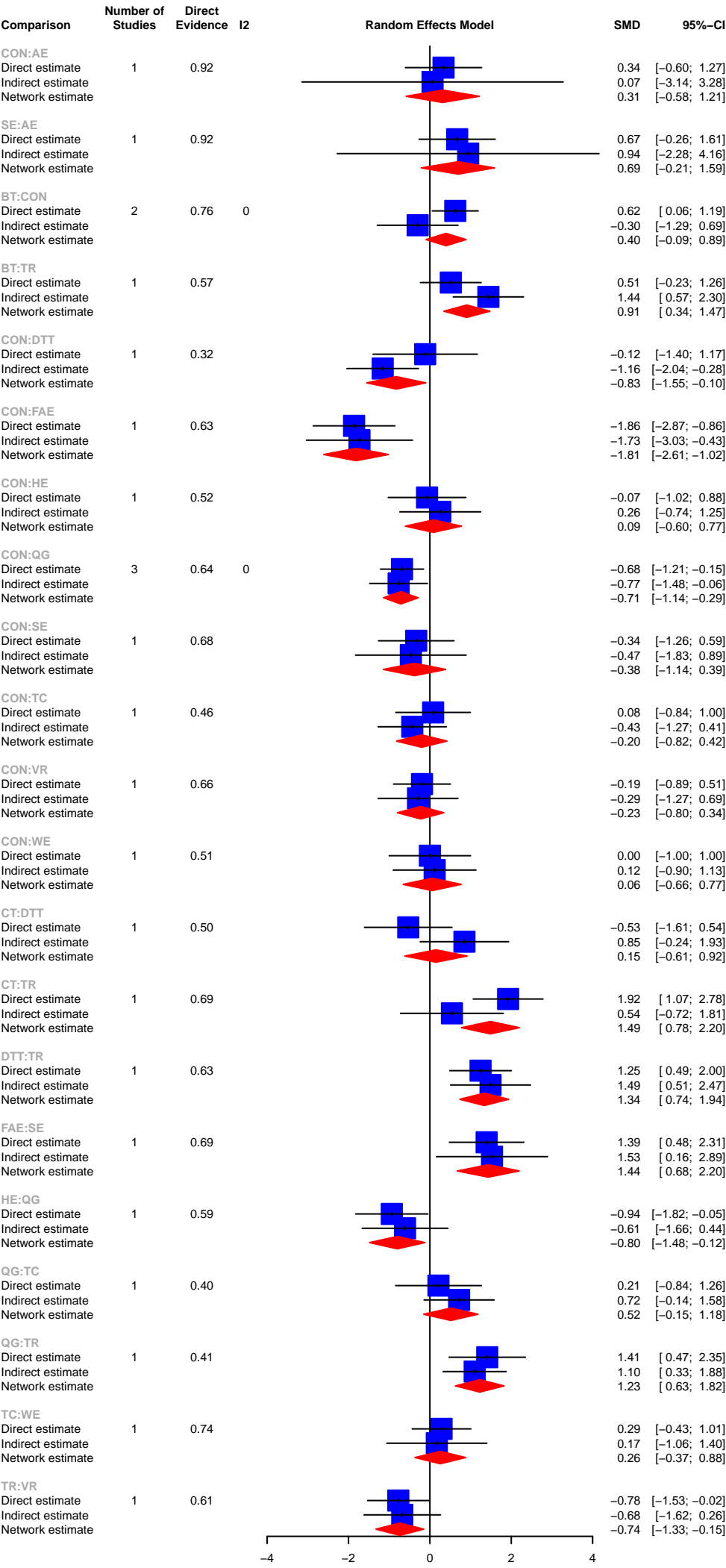

Supplement: Supplementary file 1 [file Data_Sheet_1.zip › Supplementary Material/Appendix 7.1-Node split.pdf]

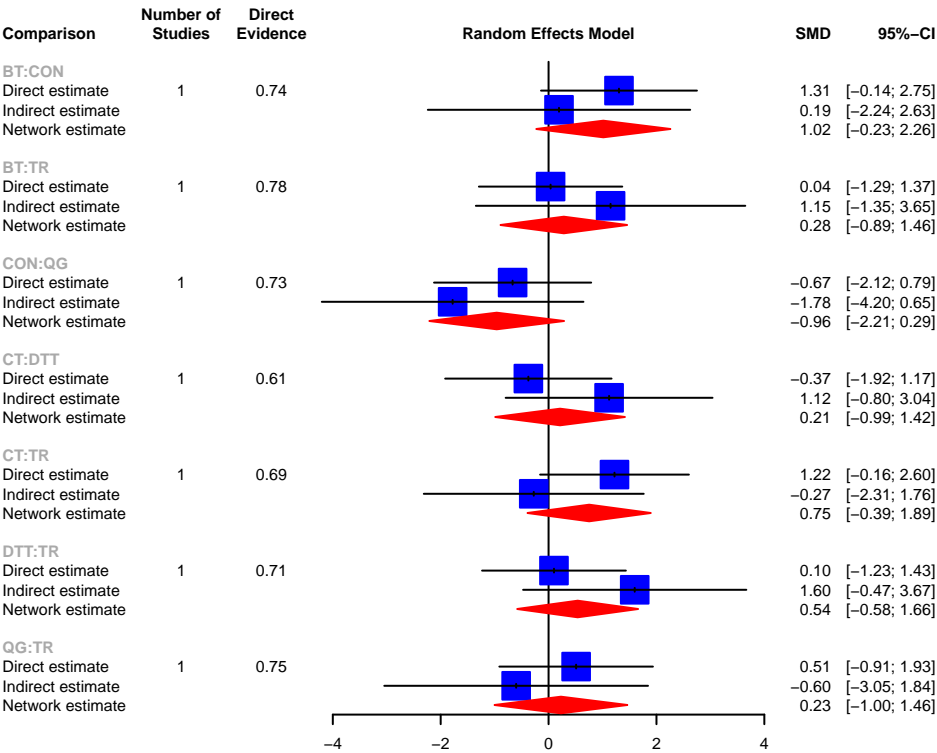

Supplement: Supplementary file 1 [file Data_Sheet_1.zip › Supplementary Material/Appendix 7.2-Node split.pdf]

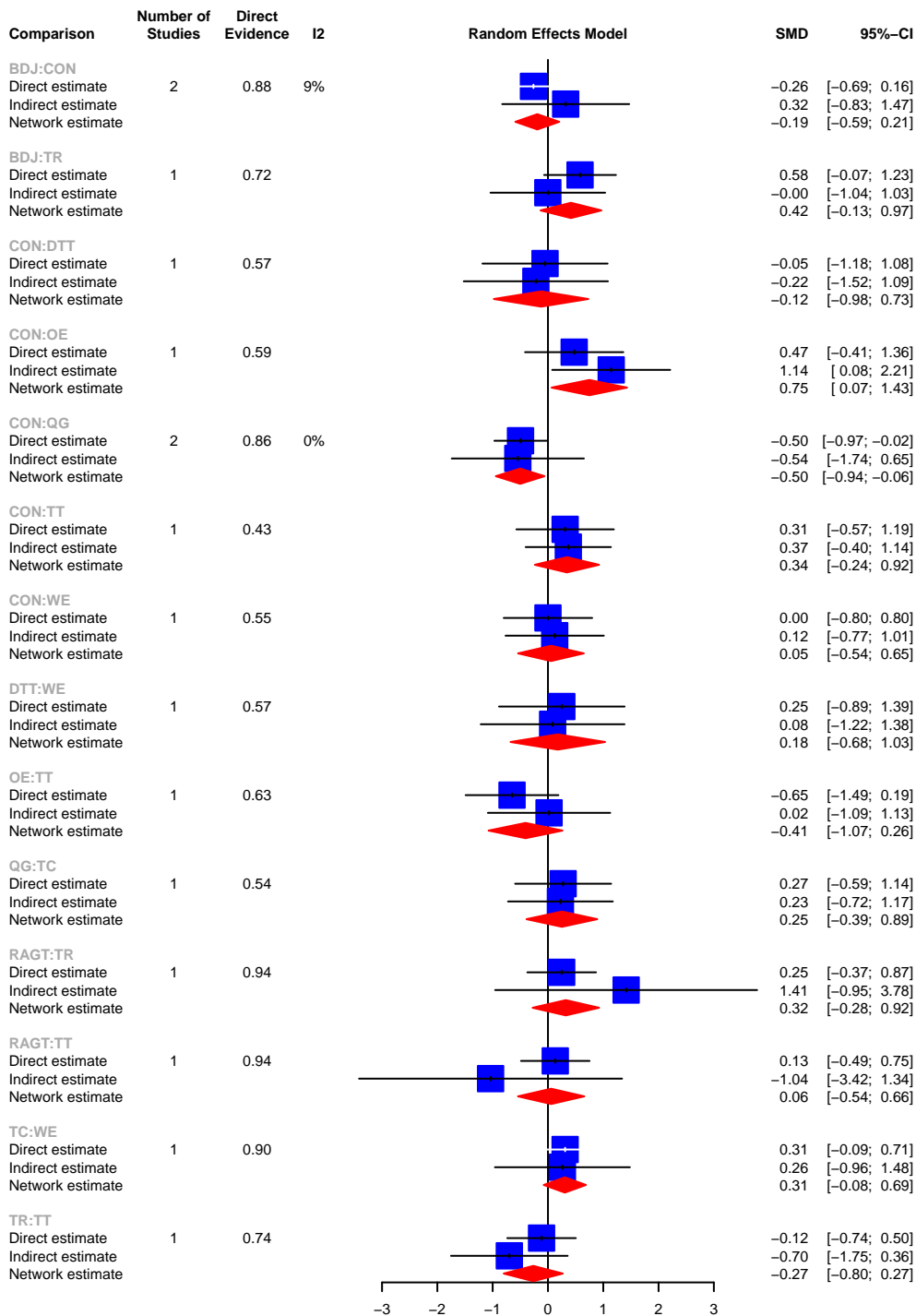

Supplement: Supplementary file 1 [file Data_Sheet_1.zip › Supplementary Material/Appendix 7.3-Node split.pdf]

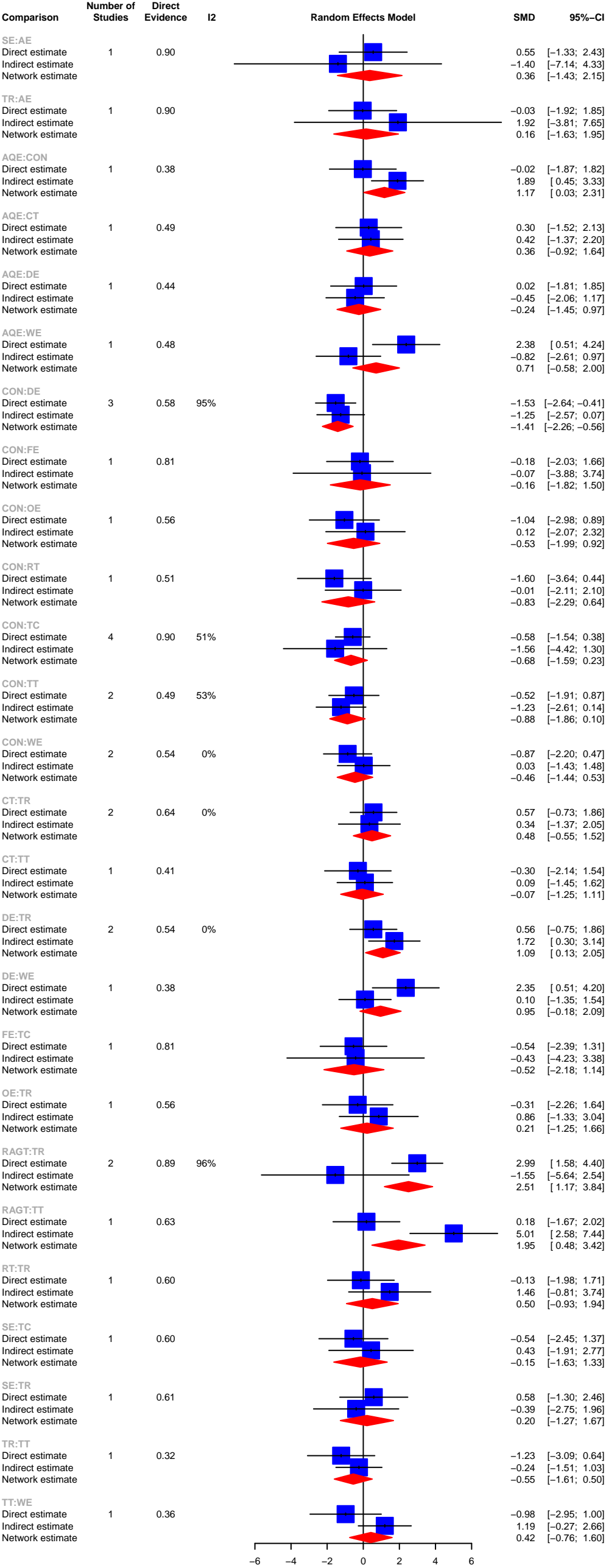

Supplement: Supplementary file 1 [file Data_Sheet_1.zip › Supplementary Material/Appendix 7.4-Node split.pdf]
